# A pilot case-control study on the fecal microbiota of pediatric functional abdominal pain-not otherwise specified and the role of early life stress

**DOI:** 10.20517/mrr.2023.75

**Published:** 2024-06-04

**Authors:** Nize Otaru, Benoît Pugin, Serafina Plüss, Iva Hojsak, Christian Braegger, Christophe Lacroix

**Affiliations:** ^1^Nutrition Research Unit, University Children’s Hospital Zürich, Zürich 8032, Switzerland.; ^2^Laboratory of Food Biotechnology, Department of Health Sciences and Technology (HEST), ETH Zürich, Zürich 8092, Switzerland.; ^3^Referral Center for Pediatric Gastroenterology and Nutrition, Children’s Hospital Zagreb, Zagreb 10000, Croatia.; ^4^University of Zagreb School of Medicine, Zagreb 10000, Croatia.; ^#^Authors contributed equally.

**Keywords:** Functional abdominal pain, functional abdominal pain not otherwise specified, fecal microbiota, fecal metabolites, early life stress

## Abstract

**Background:** Gut microbial features and the role of early life stress in pediatric functional abdominal pain-not otherwise specified (FAP-NOS) have never been investigated before. Here, we hypothesize that early life stress is more prevalent in FAP-NOS compared to healthy controls and that fecal microbial profiles and related metabolites differ between groups.

**Methods:** In an international multicenter case-control study, FAP-NOS patients (*n* = 40) were compared to healthy controls (*n* = 55). Stool samples and demographic and clinical data including early life traumatic events and antibiotics treatments were collected from children aged four to twelve years. Fecal microbial profiles were assessed with 16S rRNA gene amplicon sequencing. Microbial metabolite concentrations in fecal supernatant, including short-chain fatty acids and amino acids, were detected *via* liquid chromatography.

**Results:** Microbial richness was increased in FAP-NOS compared to healthy controls and microbial composition (unweighted UniFrac) differed between groups. Three distinct amplicon sequencing variants and two distinct species were enriched in FAP-NOS compared to controls, with no observed changes at higher taxonomic levels. No differences in microbial metabolites and early life stress were observed between groups.

**Conclusion:** The presented hypothesis could not be proven, with no observed differences in occurrence of early life stress, and fecal microbial metabolic profiles between pediatric FAP-NOS and healthy controls. Pediatric FAP-NOS patients exhibited mild differences in the fecal microbial community compared with controls. Further large-scale studies with high-resolution techniques are warranted to address the biological relevance of present observations.

## INTRODUCTION

Pediatric functional abdominal pain disorders (FAPDs), characterized by visceral hypersensitivity and dysmotility, are affecting approximately 13.5% of children worldwide, with differences observed depending on sex and country^[1,2]^. According to Rome IV criteria, they include functional dyspepsia, abdominal migraine, irritable bowel syndrome (IBS) and functional abdominal pain not otherwise specified (FAP-NOS)^[3]^. To date, diagnosis is strictly based on symptoms and exclusion of other medical conditions, with reliable biomarkers still lacking. In general, FAPDs are referred to as disorders of the gut-brain-microbiota interaction, though etiology and pathophysiology are not yet fully understood^[2-4]^. Genetic, psychosocial, and physiological factors might contribute to the disruption and/or impairment of the gut-brain-microbiota interaction. As such, risk-associated genes and altered gene expressions have been reported^[5,6]^ and a family history of FAPDs has been identified as a risk factor in adults and children alike^[2,7]^. The latter may not only result from heredity, but also from social and/or environmental factors^[2,8]^.

In early life, where infants undergo rapid development, the gut-brain-microbiota axis is thought to be especially vulnerable to perturbations^[2,9]^. Thus, early life events, ranging from childhood trauma to early life gastrointestinal infections and antibiotic treatments, have been suggested to be associated with FAPDs^[2,3,10,11]^. Increasing evidence indicates that gut microbial diversity and composition in adult functional gastrointestinal disorders, especially IBS, differ substantially from healthy controls^[12]^. Various taxa ratios, including the *Bacillota*/*Bacteroidota* and the *Faecalibacterium*/*Bacteroides* ratios*,* were previously suggested as diagnostic markers to differentiate between IBS and controls^[12,13]^. Pediatric research has also mostly been focusing on IBS, revealing increased relative abundances of genera such as *Dorea*, *Haemophilus*, and *Flavonifractor* in fecal bacterial communities of pediatric IBS patients compared to controls^[14,15]^. Additionally, a study combining metabolomics and metagenomics revealed enriched microbial metabolic pathways related to amino acid metabolism and depletion of pathways related to carbohydrate metabolism in pediatric IBS compared to controls^[15]^. Gut microbiota-derived short-chain fatty acids (SCFAs) and neuroactive compounds, such as γ-aminobutyric acid (GABA) and dopamine, are bioactive small molecules that may cross the mucosal layer and exert their effect on underlying neurons and, as such, are thought to contribute to visceral hypersensitivity in FAPDs^[2]^.

Of all FAPD subgroups, FAP-NOS and IBS are most similar, with the only discriminative criteria being the lack of association of abdominal pain with bowel movement and no change in the nature of stool in FAP-NOS^[3]^. Nevertheless, FAP-NOS and IBS have been shown to exhibit similar clinical and psychological features^[16]^. To our knowledge, there are no studies describing the gut microbial composition and associated metabolites in pediatric FAP-NOS and evaluating the occurrence of early life events (i.e., early life antibiotics treatment and traumatic events). Here, we hypothesize that the occurrence of early life events is more frequent in FAP-NOS compared to healthy controls and that fecal microbial profile and related metabolites differ distinctly between FAP-NOS and controls.

## MATERIALS AND METHODS

### Study population

In an international multicenter study, children (with FAP-NOS or healthy) between four to twelve years of age were recruited at the Children’s Hospital Zürich (Zürich, Switzerland) and Children’s Hospital Zagreb (Zagreb, Croatia). Diagnosis of children suffering from FAP-NOS was performed by pediatric specialists in accordance with Rome IV criteria^[3]^. Written informed consent was obtained from the caregivers. Ethical approval was given by the committees in Zurich (2018-00523) and Zagreb (02-23/31-2-18) for the purpose of processing biological samples and health-related data as performed in this study.

Exclusion criteria for all subjects included: (i) history of severe mental and/or psychiatric illness such as attention deficit hyperactivity disorder or autism; (ii) obesity or anorexia (BMI > P97; BMI < P3); and (iii) diabetes type 1. In addition, for healthy controls, the following exclusion criteria applied: (iv) abdominal pain; (v) intestinal infection or diarrhea in the last three months; (vi) non-regular bowel movements; (vii) intestinal diseases; (viii) lactose intolerance; and (ix) celiac disease.

Caregivers were asked about factors potentially affecting the microbiota of participants, including delivery mode, infant diet, preterm birth, current special food habits [i.e., low fermentable oligosaccharides, disaccharides, monosaccharides and polyols (FODMAP), vegetarian, vegan, gluten-free, lactose-free, or low-lactose diet], and consumption of alcohol and/or smoking. In addition, administration of loperamide, proton pump inhibitors (PPI), polyethylene glycol (PEG), probiotics, and antibiotics in the last three months was registered [Supplementary Materials]. Caregivers were asked about children’s stress in the first three years of life. As such, general early life events using the Life Event Scale^[17]^ adapted for early life, and early life antibiotics treatments were inquired [Supplementary Materials]. Early life traumatic events were assessed, using caregiver reports of the Young Children Posttraumatic Stress Disorder (PTSD) Checklist (YCPC)^[18]^ or the University of California at Los Angeles (UCLA) PTSD Reaction Index for DSM-IV^[19,20]^ modified to register the age during which specific events occurred. For subsequent data analysis, early life traumatic events were considered positive if at least one event was experienced by the participants, as indicated in YCPC or UCLA-PTSD caregiver reports in the first three years of life. Finally, FAP-NOS patients were asked to rate pain severity (from one to ten) using a visual analog scale (VAS) at the time of FAP-NOS diagnosis, and the duration of symptoms (in months) was registered.

Participants collected stool specimens at home using the provided stool catcher and sample tube. Subsequently, they stored the sample at -20 °C until transportation to research facilities (one week at the utmost). Transportation of specimens was carried out by trained professionals using a cooling container. To ensure the stability of microbial and metabolic profiles during long-term storage, the specimens were stored at -80 °C until analysis.

### Fecal dry weight and pH quantification

To determine dry weight, fecal sample aliquots of 200 mg wet weight (ww) were dried at 80 °C in a ventilated oven (VWR International AG, Dietikon, Switzerland) until stable weight was achieved (no weight change within 24 h). Fecal pH was measured as described previously^[21]^. In short, fecal slurries were prepared by homogenization of samples in double distilled water at a 1:10 ratio (ww/v) and pH was measured using a pH electrode (Metrohm Schweiz AG, Zofingen, Switzerland).

### Bacterial metabolites quantification

Fecal supernatant was prepared aerobically by homogenizing samples in 100 mM HClO_4_ at a 1:3 ratio (ww/v) followed by two centrifugation steps (6,000 × *g*, 20 min and 14,000 × *g*, 15 min, 4 °C). Supernatant samples were stored at -80 °C until analysis. Different bacterial metabolites in fecal supernatant were quantified via liquid chromatography as described previously^[22]^. In short, organic acid (i.e., SCFAs and intermediate metabolites) concentrations were analyzed directly from supernatant, while for amine, amino acid, and ammonia concentrations, a pre-column derivatization was performed prior to analysis [Supplementary Materials]. In total, 34 different metabolites were quantified, including succinate, lactate, formate, acetate, propionate, isobutyrate, butyrate, isovalerate, valerate, alanine, ammonia, arginine, asparagine, aspartic acid, cadaverine, dopamine, GABA, glutamic acid, glutamine, glycine, histidine, isoleucine, lysine, methionine, ornithine, phenylalanine/leucine, proline, putrescine, serine, tryptophan, tyramine, tyrosine, valine, and phenylethylamine. All concentrations of fecal bacterial metabolites were normalized to fecal dry weight (µmol/g).

### Metabarcoding of fecal bacterial community and enterotype assignment

Bacterial DNA was extracted from stool specimens using the FastDNA Spin kit for soil (MP Biomedicals, Illkirch, France) according to the manufacturer’s instructions.

Quantitative PCR (qPCR) was performed to assess the total 16S rRNA gene copies per sample, using primers Eub338F (5’-ACTCCTACGGGAGGCAGCAG-3’) and Eub518R (5’-ATTACCGCGGCTGCTGG-3’). Reactions were carried out in technical triplicates using the Roche Light Cycler 490 (Hoffmann-La Roche, Basel, Switzerland). Each reaction mixture consisted of 5 µL SensiFast SYBR No-ROX mix (Labgene Scientific Instruments, Châtel-Saint-Denis, Switzerland), 0.5 µL each of forward and reverse primer (10 µM, Microsynth, Balgach, Switzerland), 3 µL nuclease-free water, and 1 µL of diluted DNA template. qPCR was carried out by applying 3 min of initial denaturation at 95 °C, followed by 40 cycles of 5 seconds at 95 °C and 30 s at 65 °C. Subsequent melting curve analysis was performed from 65 to 97 °C at a ramp rate of 0.11 °C/s.

The V4 16S rRNA gene region was amplified using primers 515F (5’-GTGCCAGCMGCCGCGGTAA-3’) and 806R (5’-GGACTACHVGGGTWTCTAAT-3’), followed by tag-encoded MiSeq-based (Illumina, CA, USA) high throughput sequencing using an Illumina MiSeq System v2 including a flow cell with 2 × 250-bp paired-end Nextera chemistry supplemented with 10% of PhiX as sequencing control.

Raw Illumina sequencing data were processed using the R package metabaRpipe^[23]^. In short, Atropos^[24]^ was used to remove adaptors and V4 primers, and the DADA2 pipeline^[25]^ was used to construct Amplicon Sequencing Variants (ASV). Taxonomic assignment was performed using DADA2-formatted SILVA reference base (v138). Raw sequences were deposited on the European Nucleotide Archive at EMBL-EBI under the accession number PRJEB57328. Enterotypes were identified based on total sequences (i.e., controls and FAP-NOS). For reference-based identification of enterotypes, processed microbiota sequences were compared to a reference space of adult gut microbiota from the Human Microbiome Project (HMP)^[26]^ and Metagenomics of the Human Intestinal Tract project (MetaHIT)^[27]^ according to Arumugam *et al*.^[28]^. For *de novo* identification of enterotypes, a partitioning around medoid (PAM) clustering based on relative genus abundance using Jensen-Shannon divergence was performed^[28]^.

### Data analysis, visualization, and statistical analysis

All data and statistical analyses were performed in R (v4.2.0)^[29]^ using the packages speedyseq (v0.5.3.9018)^[30]^, vegan (v2.5.7)^[31]^, ape (v5.5)^[32]^, ampvis2 (v2.7.9)^[33]^, DivComAnalyses (v0.9)^[34]^, and Maaslin2 (v1.10.0)^[35]^. Prediction of functional metabolic potential was performed with Phylogenetic Investigation of Communities by Reconstruction of Unobserved States (PICRUSt2) software^[36]^, calculating MetaCyc pathway abundances^[37]^. Prediction accuracy was evaluated using the abundance-weighted nearest sequenced taxon index (NSTI) to summarize the extent to which ASVs in a sample are related to reference 16S rRNA genes.

For the calculation of significant differences between the two groups, a Wilcoxon rank-sum test was used for continuous variables and Fisher’s exact test was used for categorical variables. To control for false discovery rate (FDR) during multiple testing, the Benjamini-Hochberg method was applied. Fecal sample characteristics such as stool consistency (related to fecal dry weight and water content) and pH have been associated with fecal microbial features (i.e., diversity and abundance of taxa)^[38,39]^. Thus, potential confounding effects of fecal characteristics, and demographic and clinical data were addressed in multiple linear regression models [Supplementary Material and Methods] The significance level was set to *P ≤* 0.05. Data visualization was done in R using ggplot2 (v3.3.5)^[40]^ and ComplexHeatmap (v2.13.1)^[41]^. Mean or median values were stated, including standard deviation (sd) or interquartile range (iqr), in brackets, respectively. In boxplots, outliers are indicated and defined as values greater than 1.5 times the iqr over the 75th percentile and values smaller than 1.5 iqr under the 25th percentile.

## RESULTS

### Patient characteristics

In total, 123 children were recruited, of whom some were excluded due to incomplete data sets (*n* = 4), smoking (*n* = 1), detected parasites (*n* = 2), being part of siblings (*n* = 6), and antibiotics treatment in the last 6 months (*n* = 15). This resulted in 55 control and 40 FAP-NOS participants [Table 1 and Supplementary Data 1]. Age distribution significantly differed between groups, with children diagnosed with FAP-NOS being significantly older [8.6 (sd: 2.3); *P* < 0.05; FDR-adjusted] than in controls [7.1 (sd: 2.3); Table 1 and Supplementary Figure 1A]). Though not significant (*P* > 0.05; FDR-adjusted), FAP-NOS group showed a higher percentage of females (62.5%) compared to controls (56.4%). FAP-NOS patients followed special food habits significantly more often (27.5%; *P* < 0.001; FDR-adjusted) than participants of control group (0%), mainly by maintaining a lactose-free or low-lactose diet [Table 1]. Ingestion of medication such as PPI and/or PEG in the last three months was increased in FAP-NOS (15%) compared to control participants (1.8%), though this was not significant (*P* > 0.05; FDR-adjusted; Table 1). Occurrence of general early life events did not significantly differ between FAP-NOS and control groups (*P* > 0.05; FDR-adjusted**)**, with similar values for FAP-NOS and control groups [Supplementary Table 1]. Similarly, no differences in individual early life trauma events were detected between groups (*P* > 0.05; FDR-adjusted; Supplementary Table 2), with comparable occurrence of early life trauma in FAP-NOS (12.5%) and controls (12.7%; Table 1). No significant differences in early life antibiotics treatment were detected (*P* > 0.05; FDR-adjusted; Table 1), with lower trends of treatment in FAP-NOS (57.5%) compared to controls (67.3%).

**Table 1:** Group demographics and clinical data

	**Healthy controls**	**FAP-NOS**	*P*-value*
Group size (*n*)	55	40	
Age (years): mean (sd)	7.1 (2.3)	8.6 (2.3)	0.015
Sex (%): female/male	56.4/43.6	62.5/37.5	ns
Country (%): Croatia/Switzerland	50.9/49.1	27.5/72.5	ns
Mode of delivery (%): caesarian/vaginal	40.0/60.0	37.5/62.5	ns
Main infant diet in the first 4 months of life (%): breast-fed/formula-fed/both	89.1/10.9/0	70.0/22.5/7.5	ns
Preterm birth (%)	10.9	12.5	ns
Early life antibiotics treatment (in first 3 years of life; %)	67.3	57.5	ns
Early life traumatic events (in first 3 years of life; %)	12.7	12.5	ns
Probiotics treatment in last 3 months (%)	27.3	45.0	ns
Special dietary habits (%): low FODMAP/vegetarian/vegan/lactose specific/gluten-free	0/0/0/0/0	0/2.5/0/25.0/0	0.0003
Medication in last 3 months (%): PEG/PPI/loperamide	1.8/0/0	10.0/5.0/0	ns
Pain severity (VAS)^†^: mean (sd)	na	6.6 (1.9)	na
Duration of symptoms (month)^††^: mean (sd)	na	28.7 (21.0)	na

Categorical variables are expressed as the percentage of total participants per group (healthy controls *vs.* FAP-NOS). Continuous variables are expressed as mean values. Significances were calculated using the Wilcoxon rank-sum test or Fisher’s exact test including FDR correction. *FDR-adjusted; ^†^mean of 37 participants as no data available for three participants; ^††^mean of 39 participants as no data available for one participant. FAP-NOS: Functional abdominal pain-not otherwise specified; lactose specific: low lactose or no-lactose diet; ns: not significant; FODMAP: fermentable oligosaccharides, disaccharides, monosaccharides and polyols; PEG: polyethylene glycol; PPI: proton pump inhibitors; VAS: visual analog scale; sd: standard deviation; na: not applicable.

To evaluate synergistic effects of demographic and clinical data on data distribution, a multiple factor analysis was performed. This revealed a significant difference in dispersion (*P* = 0.001; R^2^ = 0.125, Supplementary Figure 1) between FAP-NOS and control groups, indicating that healthy controls are more similar amongst each other in terms of demographic and clinical data than FAP-NOS participants. In addition, the centroid distance between FAP-NOS and control groups was significant (*P* = 0.001, R^2^ = 0.135), indicating that overall demographic and clinical data of FAP-NOS are different from controls. This observed effect was mainly driven by the ingestion of medication and probiotics in the last three months, and country [Supplementary Figure 1].

### Characteristics of fecal samples

Fecal pH was identical in FAP-NOS and controls, with mean values of 7.2 (sd: 0.6) [Table 2 and Supplementary Data 1]. Fecal dry weight was similar between FAP-NOS and control groups, with approximately 30% of dry fraction in fecal samples of both groups (*P* > 0.05; FDR-adjusted; Table 2). Likewise, bacterial load - based on a total of 16S rRNA gene copies per dry weight - was comparable between groups, with mean values of 10^12^ copies for both groups [Table 2]. To evaluate a potential difference in general microbiota type between groups, identification of enterotypes was performed. Reference-based enterotype assignment resulted in 67% of samples showing major compositional dissimilarity to the reference space based on the adult microbiota of HMP^[26]^ and MetaHIT^[27]^. Therefore, *de novo* enterotype assignment was performed. Two distinct enterotypes were identified, (i) enterotype 1 enriched in 12 distinct genera and (ii) enterotype 2 enriched in 20 distinct genera [Supplementary Figure 2], with no significant differences between FAP-NOS and control participants in terms of enterotype distribution (*P* > 0.05; FDR-adjusted; Table 2). Enterotype 1 showed a high abundance of *Collinsella* species (5.15%; iqr: 5.36%) while enterotype 2 showed a high abundance of *Bacteroides* species (18.36%; iqr: 7.52%; Supplementary Figure 3). No associations of different enterotypes and bacterial-produced organic acids were detected [Supplementary Figure 4]. However, enterotype 1 was characterized by increased fecal amine and amino acid concentrations, including alanine, cadaverine, glycine, isoleucine, phenylalanine/leucine, proline, tryptophan, tyrosine, and valine [Supplementary Figure 5]. In addition, pH values for enterotype 1 were significantly lower (*P* < 0.001), though with a weak effect size (r = 0.270) and median values of 7.1 (iqr: 0.8) and 7.5 (iqr: 0.8) for enterotype 1 and enterotype 2, respectively [Supplementary Figure 6]. Enterotypes 1 and 2 exhibited different correlation profiles between significantly enriched genera and metabolites [Supplementary Figure 7].

**Table 2:** Characteristics of fecal samples

	**Healthy controls**	**FAP-NOS**	*P*-value*
Fecal pH: mean (sd)	7.2 (0.6)	7.2 (0.6)	ns
Fecal dry weight (%): mean (sd)	27.9 (6.8)	28.4 (6.9)	ns
Log_10_ 16S rRNA gene copies per g dry weight: mean (sd)	12.05 (0.40)	11.86 (0.43)	ns
*De novo* enterotype (%): enterotype 1/enterotype 2	60/40	75/25	ns

Categorical variables are expressed as percentages of total participants per group (healthy controls *vs.* FAP-NOS). Continuous variables are expressed as mean values. Significances were calculated using the Wilcoxon rank-sum test or Fisher’s exact test including FDR correction. *FDR-adjusted. FAP-NOS: Functional abdominal pain-not otherwise specified; sd: standard deviation; ns: not significant

### Increased fecal bacterial richness in FAP-NOS group compared to controls

Alpha diversity - diversity within the community - was investigated at the ASV and species levels via evaluation of gut microbiota richness (observed ASVs or observed species; number of ASVs or species in a sample), and evenness (Pielou’s index, a measure indicating how evenly ASVs or species are distributed in a sample). Potential confounding effects of demographic and clinical data [Table 1], and fecal characteristics [Table 2] on alpha diversity were tested in a multiple linear regression model.

**Figure 1:**
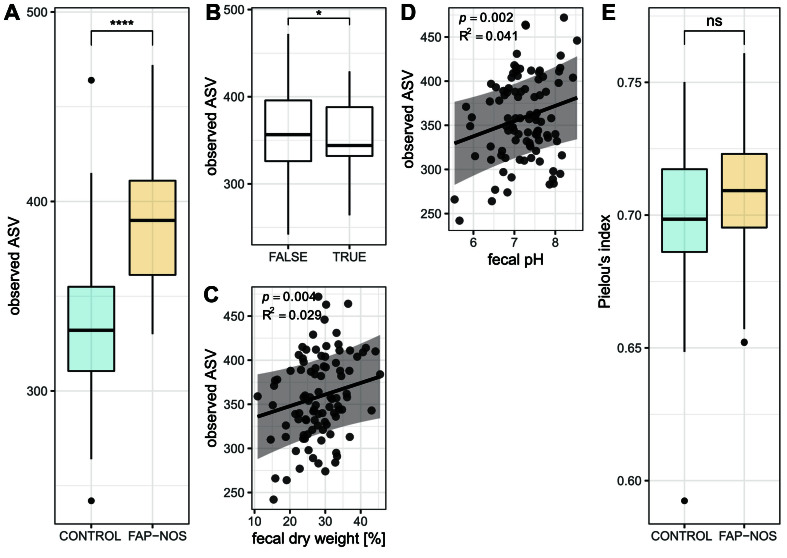
Alpha diversity at ASV level. Comparison of (A) richness and (E) evenness between FAP-NOS and control groups; Visualization of the effect of (B) probiotics treatment in the last three months (FALSE *vs.* TRUE); (C) fecal dry weight; and (D) fecal pH on gut microbial richness. Boxplot with box elements showing upper quantile, lower quantile, and median. Whiskers extend from the upper/lower quantile to ± 1.5 iqr range or the highest/lowest value. Outliers are indicated as black points. Points in the scatterplot display values for individual microbiota. Regression line is based on multiple linear regression model, and 95% confidence interval is displayed. All significances were calculated using a multiple linear regression model [Supplementary Table 3]. *P < 0.05; ****P < 0.0001. FAP-NOS: Functional abdominal pain-not otherwise specified; ns: not significant; ASV: amplicon sequence variant; iqr: interquartile range.

At the ASV level, gut microbiota richness was significantly higher in FAP-NOS patients compared to controls (*P* < 0.0001; R^2^ = 0.263), with medians of observed ASVs being 390 (iqr: 50) and 332 (iqr: 45), respectively [Figure 1A]. Similarly, probiotics treatment in the last three months significantly (*P* < 0.05; R^2^ = 0.029) decreased richness, with medians of observed ASVs being 344 (iqr: 56) and 357 (iqr: 70) for probiotic and non-probiotic, respectively [Figure 1B]. Fecal dry weight (*P* < 0.05; R^2^ = 0.029; Figure 1C) and pH (*P* < 0.05; R^2^ = 0.041; Figure 1D) both positively correlated with richness. No other demographic and clinical data, and fecal characteristics explained a significant amount of variation in gut bacterial richness (*P* > 0.05, Supplementary Table 3). Pain severity and duration of symptoms did not correlate (*P* > 0.05) with gut microbiota richness in FAP-NOS patients. No significant difference between FAP-NOS and controls (*P* > 0.05) in gut microbiota evenness was detected, with a comparable median Pielou’s index of 0.70 (iqr: 0.03) for both groups [Figure 1E]. No effects of demographic and clinical data, and fecal characteristics on gut microbiota evenness were observed.

Similar results were observed at the species level, with higher richness in FAP-NOS compared to controls (*P* < 0.0001; R^2^ = 0.174), and no differences observed for gut microbiota evenness (*P* > 0.05) [Supplementary Figure 8]. However, at the species level, no effects of underlying demographic and clinical data, and fecal characteristics on gut microbiota richness were observed [Supplementary Table 4].

### Mild differences in fecal microbial composition between FAP-NOS and control groups

Beta diversity - diversity between communities – was investigated at the ASV and species levels via evaluation of the UniFrac distance. This beta diversity metric includes phylogenetic (i.e., evolutionary relationship) information. Unweighted (qualitative) UniFrac distance served as a read-out for gut microbial composition, and weighted (quantitative) UniFrac distance served as a read-out for gut microbiota structure. Potential confounding effects of demographic and clinical data [Table 1], and fecal characteristics [Table 2] on beta diversity were addressed in a linear model fitted to various distance metrics.

**Figure 2:**
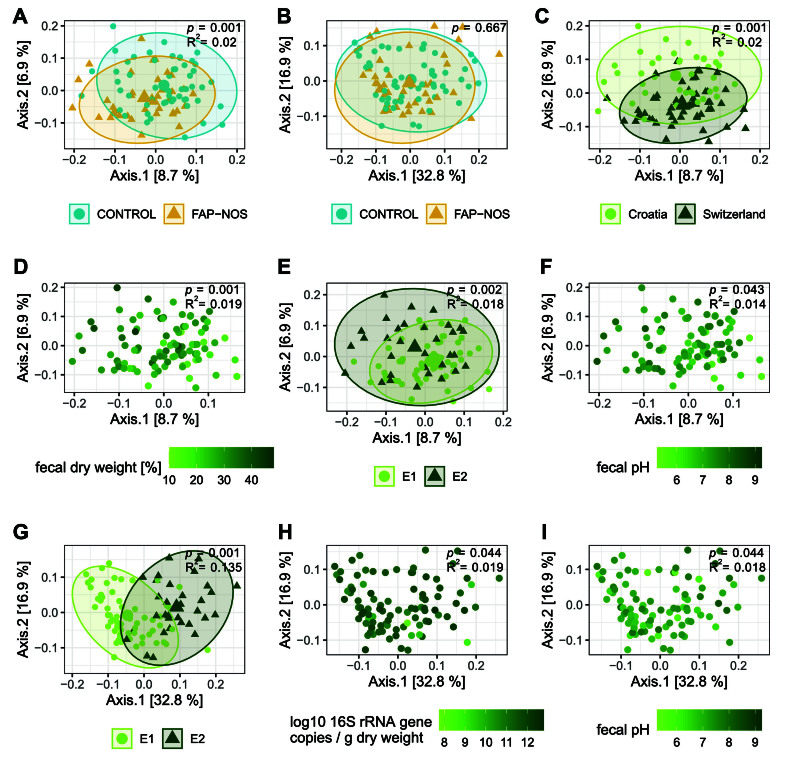
Beta diversity metrics at ASV level. Comparison of (A) unweighted and (B) weighted UniFrac between FAP-NOS (orange triangle) and control groups (blue circle); Visualization of effect of (C) country, (D) fecal dry weight, (E) enterotype, and (F) fecal pH on unweighted UniFrac; Visualization of effect of (G) enterotype, (H) bacterial load, and (I) fecal pH on weighted UniFrac. Small symbols display individual microbiota, large symbols display centroids, and ellipses indicate 95% confidence intervals. All significances were calculated using a linear model fitted to various distance metrics [Supplementary Tables 5 and 6]. FAP-NOS: Functional abdominal pain-not otherwise specified; E1: enterotype 1; E2: enterotype 2; ASV: amplicon sequence variant.

At the ASV level, gut microbiota composition based on unweighted UniFrac was significantly different between FAP-NOS and controls (*P* = 0.001), though with low explanatory power (R^2^ = 0.020; Figure 2A). Country, *de novo* enterotype, fecal dry weight, and fecal pH all explained a similar amount of variability observed in unweighted UniFrac (0.014 ≤ R^2^ ≤ 0.020; Figure 2 and Supplementary Table 5). Gut microbiota structure based on weighted UniFrac did not differ significantly between FAP-NOS and control groups (*P* > 0.05; Figure 2B). The variability observed in weighted UniFrac was mainly explained by different *de novo* enterotypes (*P* = 0.001; R^2^ = 0.135; Figure 2G) and, to a lesser extent, by bacterial load (*P* < 0.05; R^2^ = 0.019; Figure 2H) and fecal pH (*P* < 0.05; R^2^ = 0.018; Figure 2I and Supplementary Table 6). Pain severity and duration of symptoms did not explain a significant amount of variability observed in both beta diversity metrics (*P* > 0.05).

Similar results were observed at the species level, with significant differences between FAP-NOS and controls for unweighted UniFrac (microbiota composition; *P* < 0.002; R^2^ = 0.029) and no differences in weighted UniFrac (microbiota structure; *P* > 0.05; Supplementary Figure 9). However, at the species level, fecal dry weight, fecal pH, and bacterial load had no effect on observed variability [Supplementary Figure 9, Supplementary Tables 7 and 8].

To further investigate microbial composition and structure, common taxa ratios were explored and fitted to various demographic and clinical data, and fecal characteristics. Both the *Bacillota*/*Bacteroidota* and the *Faecalibacterium*/*Bacteroides* ratio did not significantly differ between FAP-NOS and control groups (*P* > 0.05), with comparable values for both [Supplementary Figures 10A and 11A]. However, *de novo* enterotypes and fecal pH or *de novo* enterotypes, infant diet and fecal dry weight significantly affected the *Bacillota*/*Bacteroidota* or the *Faecalibacterium*/*Bacteroides* ratio, respectively [Supplementary Figure 10B-D, Supplementary Tables 9 and 10]. Pain severity and duration of symptoms did not correlate with the *Bacillota*/*Bacteroidota* or the *Faecalibacterium*/*Bacteroides* ratio (*P* > 0.05).

**Figure 3:**
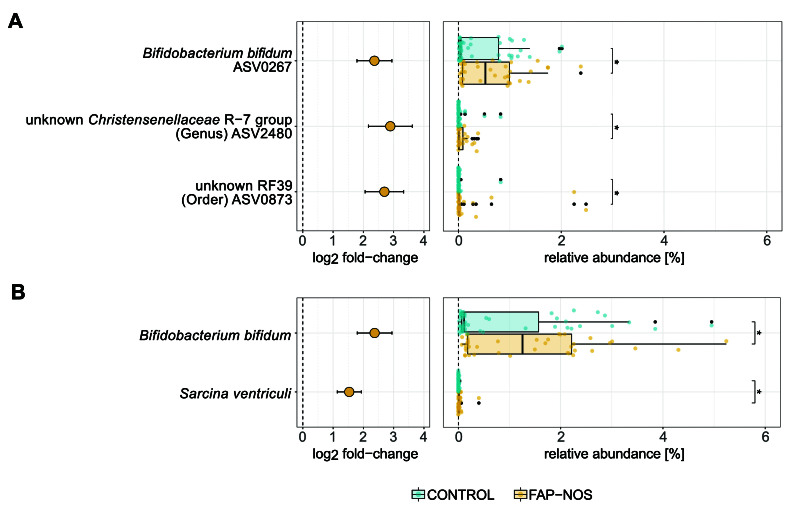
Differential abundance of ASVs and species in FAP-NOS *vs.* controls. Abundance log_2_ fold-changes and relative abundance of significantly (*P* < 0.05) increased (A) ASVs and (B) species in FAP-NOS compared to controls. Points display individual log_2_ fold-changes and bars display standard errors. Boxplot with box elements showing upper quantile, lower quantile, and median. Whiskers extend from the upper/lower quantile to ± 1.5 iqr or the highest/lowest value. Colored points display values for individual microbiota, while outliers are indicated as black points. Significances were calculated using a log_2_ transformed linear model with FDR correction for multiple testing. *P < 0.05. FAP-NOS: Functional abdominal pain-not otherwise specified; ASVs: amplicon sequence variants; iqr: interquartile range; FDR: false discovery rate.

To identify specific taxa that are differentially abundant in FAP-NOS compared to controls, a differential abundance analysis was performed by applying a linear model. Three significantly (*P* < 0.05; FDR-adjusted) differential abundant ASVs between FAP-NOS and controls were detected, and all increased in FAP-NOS in the range of 2 to 3 log_2_ fold-changes. The relative abundance of these ASVs was low, with individual microbiota values ranging between 0% and 2.5% [Figure 3A]. ASV0873, belonging to an unknown species of the RF39 order, was present in 36.7% and 65.0%, and ASV2480, belonging to an unknown species of the genus *Christensenellaceae* R-7 group, was present in 34.5% and 72.5% of control and FAP-NOS samples, respectively. ASV0267, identified as a *Bifidobacterium bifidum* ASV, was present in all tested samples.

*Sarcina ventriculi* and *Bifidobacterium bifidum* were both significantly (*P* < 0.05; FDR-adjusted) increased in FAP-NOS compared to controls, with observed log_2_ fold-changes of 1.5 and 1.4, respectively. Overall, the relative abundance of *Sarcina ventriculi* was low, with the highest observed value of 0.4% [Figure 3B]. *Sarcina ventriculi* was present in 16.4% of control and 57.5% of FAP-NOS samples, while *B. bifidum* was present in all tested samples. The highest observed abundance of *B. bifidum* was 5.2%, with median values of 1.3% (iqr: 2.0) and 0.1% (iqr: 1.5) for FAP-NOS and controls, respectively.

**Figure 4:**
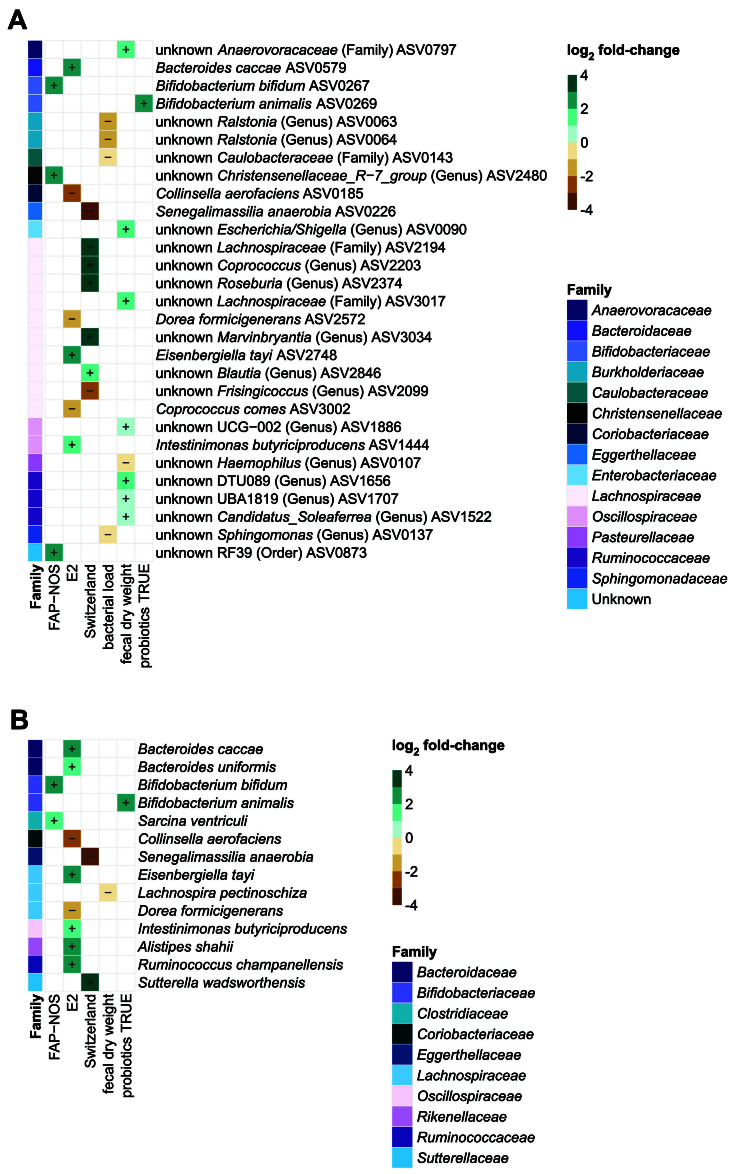
Differential abundance of ASVs and species in demographic and clinical data and fecal characteristics. Heatmaps of all significantly (*P* < 0.05) differentially abundant (A) ASVs and (B) species in demographic and clinical data and fecal characteristics. Log_2_ fold-change per unit of continuous variables or compared to a corresponding categorical variable is displayed. Additional taxonomic information is indicated at the family level. Significances were calculated using a log_2_ transformed linear model with FDR correction for multiple testing. FAP-NOS: Functional abdominal pain-not otherwise specified; E2: enterotype 2; ASVs: amplicon sequence variants; FDR: false discovery rate.

No significantly differentially abundant taxa (*P* > 0.05; FDR-adjusted) between FAP-NOS and controls were identified at higher taxonomic levels [Supplementary Data 2]. The abundance of ASVs and/or species was different when comparing *de novo* enterotypes (E1 *vs.* E2), or countries (Switzerland *vs.* Croatia), or probiotics treatment status (probiotic intake: FALSE *vs.* TRUE) [Figure 4]. Bacterial load and fecal dry weight both negatively correlated with specific ASVs or species, respectively [Figure 4]. Pain severity and duration of symptoms did not correlate with abundance of any taxa (*P* > 0.05; FDR-adjusted). A subsequent analysis of FAP-NOS microbiota alone was performed to examine the potential effect of age, given the broad age range in the present study. No significant effects (*P* > 0.05) of age on taxa abundances at genus and family level were observed.

### No differences in fecal microbial metabolites between FAP-NOS and control groups

The metabolic profiles of FAP-NOS and controls [Supplementary Data 3] were evaluated and compared under the inclusion of various demographic and clinical data and fecal characteristics. No significant differences (*P* > 0.05; FDR-adjusted) between FAP-NOS and controls in fecal total and specific organic acid, amine, amino acid, and ammonia concentrations were detected, including 34 different metabolites, i.e., succinate, lactate, formate, acetate, propionate, isobutyrate, butyrate, isovalerate, valerate, alanine, ammonia, arginine, asparagine, aspartic acid, cadaverine, dopamine, GABA, glutamic acid, glutamine, glycine, histidine, isoleucine, lysine, methionine, ornithine, phenylalanine/leucine, proline, putrescine, serine, tryptophan, tyramine, tyrosine, valine, and phenylethylamine [Supplementary Figures 12 and 13]. However, country, fecal dry weight, fecal pH, *de novo* enterotype, and main infant diet had a significant effect on the fecal metabolite profile [Supplementary Figure 14].

To further elaborate on fecal metabolic potential, a preliminary explorative prediction of bacterial metabolic pathways based on 16S rRNA microbial profiling was performed [Supplementary Data 4]. Abundance-weighted average NSTI scores were not significantly different between FAP-NOS and controls (*P* > 0.05). Individual microbiota values ranged from 0.03 to 0.18, indicating that, on average, prediction was performed based on taxa with 97% to 82% similarity. In total, 349 distinct metabolic pathways were identified. FAP-NOS and control groups did not significantly differ (*P* > 0.05; FDR-adjusted) in any predicted metabolic pathways. Significantly differentially abundant metabolic pathways were observed between different *de novo* enterotypes. Fecal dry weight, fecal pH, and bacterial load significantly correlated with different metabolic pathways [Supplementary Figure 15]. Pain severity and duration of symptoms did not correlate with abundance of any pathway (*P* > 0.05; FDR-adjusted).

## DISCUSSION

Of all FAPD subgroups, FAP-NOS is the least characterized, and associated biomarkers have never been reported before. In this pilot-case-control study, for the first time, the microbial community and associated metabolites in pediatric FAP-NOS patients are addressed and the occurrence of early life events - defined as early life traumatic events and antibiotics treatment - is evaluated.

Early life stress has previously been suggested as a risk factor for the development of FAPDs, as the first years of life pose a critical developmental window. Early life events may thus have lasting consequences and may alter pain processing later in life^[2,9,42]^. On the contrary, here we showed no differences in experienced traumatic early life events and early life antibiotics treatment in FAP-NOS compared to controls. In addition, no significant associations of traumatic early life events and/or early life antibiotics treatment with fecal microbial diversity, composition, and structure, including associated metabolites, were observed. However, we repeatedly show a significant effect of country, i.e., Switzerland *vs.* Croatia, and fecal characteristics, i.e., fecal pH, fecal dry weight, bacterial load, and *de novo* enterotype, on the fecal bacterial community. Contrary to previously observed three enterotypes in school-aged children, dominated by *Bacteroides*, *Prevotella* or *Bifidobacterium*^[43]^, and enterotypes identified in adults i.e., *Bacteroides*, *Prevotella* or *Ruminococcus* dominated^[44]^, here we identified only two *de novo* enterotypes enriched in 12 or 20 distinct genera with concurring difference in metabolic profiles and predicted metabolic pathways. More specifically, enterotype 2 was enriched in *Bacteroides* and *Alistipes* species, which have previously been associated with amino acid degradation^[45,46]^. In line, enterotype 2 was depleted in amino acids (among others, valine, isoleucine, proline, and alanine) compared with enterotype 1, and depleted in predicted metabolic pathways for valine and isoleucine biosynthesis. Concurringly, for enterotype 2, the genus *Bacteroides* significantly negatively correlated with alanine and proline, while the genus *Alistipes* significantly negatively correlated with proline concentrations.

We showed a significant increase in microbiota richness in FAP-NOS compared to controls while controlling for demographic and clinical data, and fecal characteristics. Previous research has illustrated the increase in microbial richness with increasing luminal pH^[39]^, which we confirm here. However, precise mechanisms of pH on microbial features are not yet known. In the adult population, microbiome richness is associated with health due to improved community resilience promoted by increased efficiency and redundancy^[47]^, while decreased richness is associated with various diseases (e.g., diabetes, inflammatory bowel diseases, obesity)^[48,49]^. In the rarely available studies referring to pediatric FAPDs patients, inconsistent results were reported, with microbiota alpha diversity metrics in patients being increased, decreased or similar compared to the controls^[13,14,50]^. These inconsistent results might be explained by different FAPD subgroups, distinct dietary preferences, e.g., high fiber intake^[51]^, or by potential underlying microbiota confounders. Indeed, a strong association linking increased richness with low *Bacteroidota* and high *Bacillota* abundance was previously shown^[52]^. Nevertheless, in our study, the *Bacillota*/*Bacteroidota* ratio showed no significant difference between FAP-NOS and controls, thereby further strengthening the differences in alpha diversity observed.

Multi-omics studies in pediatric and adult IBS patients alike suggest a correlation between microbial features and symptom severity^[15,53]^. However, no correlation between pain intensity and duration of symptoms with fecal microbial community and associated metabolites was observed in the present study. We showed a significant difference in fecal bacterial composition (unweighted UniFrac) and no difference in fecal bacterial structure (weighted UniFrac) between FAP-NOS and healthy controls. However, the degree of variation explained in bacterial composition was low and similar to that of the country, *de novo* enterotype, fecal dry weight, and fecal pH, thus questioning the clinical relevance of those differences as biomarkers. The *Faecalibacterium/Bacteroides* ratio has previously been suggested as a biomarker for FAPDs, with lower ratios observed in FAPDs patients^[13]^. However, in the present study, no difference in this taxa ratio was observed between FAP-NOS and controls. Instead, we showed enrichment of *Sarcina ventriculi* and *B. bifidum* in pediatric FAP-NOS compared to controls, with no observed changes at higher taxonomic levels. *Sarcina ventriculi* is a gut commensal found in fecal samples of healthy adults mainly following a vegetarian diet^[54]^. However, the extent of pathogenicity of *Sarcina ventriculi* is still discussed. In rare cases, *Sarcina ventriculi* infections have been reported, with the main isolation location being the gastroesophageal tract. Interestingly, abdominal pain was one of the major comorbidities observed^[55]^. In general, high counts of *Bifidobacterium* species are associated with healthy microbiota and probiotic formulations containing specific strains of this group have also been suggested as a treatment for pediatric IBS, alleviating abdominal pain^[56,57]^. In adult IBS patients, treatment with a specific *B. bifidum* strain resulted in a significant decrease in pain symptoms compared to placebo group^[58]^. However, the sequence similarity of the 16S rRNA genes of some *Bifidobacterium* groups has been described as being exceptionally high (99% similarity)^[59,60]^. Together with the notion that the V4 region of the 16S rRNA gene may not always be able to differentiate between species^[61]^, caution is required when interpreting these results. We show an association between probiotic intake in the last three months and an increase in *B. animalis* in overall fecal microbiota. Given the high sequence similarities of the 16S rRNA genes and the higher, though non-significant, probiotic intake in the FAP-NOS group, an influence of probiotics on the observed results of *Bifidobacterium* abundances cannot be excluded. High-resolution techniques, such as shotgun metagenomic sequencing, are thus warranted to further shed light on specific *Bifidobacterium* species changes between FAP-NOS and healthy controls. Given the lack of differences in bacterial abundance at higher taxonomic levels (i.e., genus and family level) and the relatively small amount (2%) of variation explained by health status (i.e., FAP-NOS *vs.* control), our data suggest mild alterations in FAP-NOS microbiota compared to controls.

Here, we showed that fecal organic acid and amino acid composition, including SCFAs and neuroactive compounds, did not significantly differ between FAP-NOS patients and healthy children. In addition, no differences were observed in predicted bacterial metabolic pathways. Similarly, a study comparing volatile organic acids in fecal samples from pediatric IBS, FAP-NOS and controls did not show differences between groups^[62]^. Nevertheless, a multi-omics study in pediatric IBS patients revealed a trend toward a decrease or increase in predicted metabolic pathways and enzymes involved in microbial carbohydrate degradation or amino acid metabolism, respectively^[15]^. In addition, formate, lactate, tyrosine, lysine, and leucine have been shown to be enriched in pediatric IBS patients compared to healthy controls^[63]^. Given that before-mentioned studies focus on diarrhea or constipation-predominant IBS with associated differences in colon transit time and thus fermentation time, it seems plausible that no effects are seen in FAP-NOS where altered colon transit time is not present^[3]^. The metabolic pathway prediction with PICRUSt2 has limitations, such as bias resulting from a strong dependency on reference genomes and the inability to address strain-specific functionality^[36]^. Thus, the present PICRUSt2 analysis is a preliminarily explorative prediction and would need to be verified with shotgun metagenomics, metabolomics, and/or RNA sequencing. Specific alterations of gut microbial functionality in FAP-NOS patients may be revealed only when high-resolution techniques are applied.

It must be noted that the present study included children from a relatively wide age range of 4 to 12 years. Based on fecal microbial richness, previous research has suggested that the maturation of the gut microbiota extends beyond the fifth year of life^[64]^, suggesting potential differences between different ages in the present study. Even though we show a significant difference in terms of age between children recruited for the control and FAP-NOS groups, in subsequent linear regression models, there was no association between age and different microbial features (i.e., alpha and beta diversity, taxa abundances, metabolites, and predicted metabolic pathways). In addition, no age-related variations in FAP-NOS patients were observed.

The present study has several limitations. Firstly, although fecal samples have their advantages as a non-invasive form of investigation of the gut microbiota, they serve only as a proxy of the gut microbial community present in different regions of the colon and its metabolic potential^[65]^. In association with nutrient availability (carbohydrates *vs.* proteins) and pH gradient, the gut microbial community and functionality differ across the lateral (mucosa to lumen) and longitudinal (proximal to distal) axis of the colon^[66]^. Observed changes in fecal microbial community or lack thereof may thus not be directly extrapolated to the entire gut microbial community. Thus, the biological relevance of observed changes needs to be addressed in future studies. Secondly, the present study is a pilot study with a relatively small sample size. Only a large-scale study would allow for further stratification of the heterogenous FAP-NOS group. Even though the present study addressed various potential confounders, Falony et. al have shown that up to 69 clinical and questionnaire-based covariates could be associated with the gut microbial composition of healthy individuals^[67]^. Likewise, the fecal metabolome is affected by various environmental and medical factors such as physical fitness and specific diets^[68,69]^. Future studies should thus address additional confounders such as detailed food habits. In the present study, early life traumatic events were evaluated using validated PTSD caregiver reports. However, there are less obvious experiences, such as unstable families or poverty, that pose a substantial burden on children. In general, there is still considerable ambiguity concerning the definition of early life stress itself and aspects including parental care or dysfunctional relationship between parents and children may not be captured by caregiver reports^[70]^.

Conclusively, no differences in the occurrence of early life stress and fecal metabolic profiles between FAP-NOS and controls were observed; thus, the present hypothesis could not be verified. Yet, the present study investigated for the first time the fecal microbial community in pediatric FAP-NOS, revealing significant differences between FAP-NOS and healthy children. The observed microbial profile was different from previously described changes in pediatric IBS. Specifically, we showed an increased fecal microbial richness in FAP-NOS compared to controls, and significant yet mild alterations in fecal microbial composition (unweighted UniFrac), including three distinct ASVs and two distinct species. Further large-scale studies with high-resolution techniques are warranted to confirm present observations and further address potential microbial functional differences in FAP-NOS and controls.

## DECLARATIONS

### Availability of data and materials

Raw sequences generated and analyzed during the current study are available in the European Nucleotide Archive repository under accession number PRJEB57328 (https://www.ebi.ac.uk/ena/browser/view/PRJEB57328).

### Copyright

© The Author(s) 2024.
