# Supplementary material for: A pilot case-control study on the fecal microbiota of pediatric functional abdominal pain-not otherwise specified and the role of early life stress
Source: Microbiome Res Rep. 2024 Jun 4;3(3):32. doi: 10.20517/mrr.2023.75 (PMC11485736; doi:10.20517/mrr.2023.75)
Supplement: Supplementary file 1 [file mrr-3-3-32-SupplementaryMaterials.zip › mrr2075-SupplementaryMaterials/Supplementary Materials.pdf]

Supplementary Materials

A pilot case-control study on the microbiota of pediatric functional abdominal pain-not otherwise specified and the role of early life stress

Nize Otaru[1,2], Benoît Pugin[2], Serafina Plüss[2], Iva Hojsak[3,4], Christian Braegger[1,#], Christophe Lacroix[2,#]

[1]Nutrition Research Unit, University Children's Hospital Zürich, Zürich 8032, Switzerland.
[2]Laboratory of Food Biotechnology, Department of Health Sciences and Technology (HEST), ETH Zürich, Zürich 8092, Switzerland.
[3]Referral Center for Pediatric Gastroenterology and Nutrition, Children's Hospital Zagreb, Zagreb 10000, Croatia.
[4]University of Zagreb School of Medicine, Zagreb 10000, Croatia.
[#]Authors contributed equally.

Correspondence to: Prof. Christophe Lacroix, Laboratory of Food Biotechnology, Department of Health Sciences and Technology (HEST), Schmelzbergstrasse 7, LFV C20, ETH Zürich, Zürich 8092, Switzerland. E-mail: christophe.lacroix@hest.ethz.ch

Supplementary Material and Methods

Fecal bacterial metabolites quantification

Bacterial metabolites in fecal supernatant samples were quantified as described previously[17]. In short, for quantification of organic acids (i.e., SCFAs and intermediate metabolites) 200 µL of supernatant sample was passed through a 0.45 µm nylon membrane filter. Subsequent separation was performed on a LaChrom HPLC-System (Merck-Hitachi, Japan) using a SecurityGuard Cartridges Carbo-H (4 × 3.0 mm; Phenomenex Inc., Torrance, CA, United States) connected to a Rezex ROA-Organic Acid H+ (300 × 7.8 mm; Phenomenex Inc.) column, with an injection volume of 40 µL. Elution of samples was carried out at 40 °C under isocratic conditions (10 mM H_2SO_4) and a flow rate of 0.4 mL/min. Analytes were quantified using a refractive index detector L-2490 (Merck Hitachi) and data was processed using the EZChrom software (Agilent, Santa Clara, CA, United States).

For amine, amino acid and ammonia concentrations a pre-column derivatization was performed by mixing 100 µL of supernatant sample with 175 µL borate buffer (1 M H_3BO_3 adjusted to pH 9 with NaOH), 75 µL methanol, 4 µL internal standard (2 g/L L-2-aminoadipic acid, Sigma-Aldrich Chemie GmbH, Buchs, Switzerland), and 3.5 µL diethyl ethoxymethylenemalonate (VWR International AG, Dietikon, Switzerland). The mixture was incubated at room temperature in an ultrasonic bath for 45 min, and subsequently heated at 70 °C for 2 h to stop the reaction. Resulting samples were passed through a 0.2 µm nylon membrane filter. Subsequent separation was performed on an ACQUITY UPLC H-Class System (Waters Corp., Milford, MA, United States) using an ACQUITY BEH C18 column (1.7 µm particle size; 2.1 × 100 mm; Waters Corp.), with an injection volume of 1 µL. Elution of samples was performed at 40 °C and a flow rate of 0.46 mL/min, using a gradient of (A) 25 mM acetate buffer (pH 6.6), (B) 100% methanol, and (C) 100% acetonitrile: 0-2 min [A 92%-93%, B 2%-1.5%, C 6%-5.5%]; 2-4.5 min [A 93%-85%, B 1.5%-4%, C 5.5%-11%]; 4.5-6.5 min [A 85%, B 4%, C 11%]; 6.5-8 min [A 85%-80%, B 4%-6%, C 11%-14%], 8-12.5 min [A 80%-70%, B 6%-2%, C 14%-28%]; 12.5-15.5 min [A 70%-55%, B 2%-3%, C 28%-42%]; 15.5-18 min [A 55%-45%, B 3%-1%, C 42%-54%]; 18-20 min [A 45%-0%, B 1%-20%, C 54%-80%]; 20-27 min [A 0%, B 20%, C 80%]; 27-28 min [A 0%-90%, B 20%-2%, C 80%-8%]; and 28-30 min [A 90%, B 2%, C 8%]. Analytes were quantified using a diode array detector at 280 nm. Raw data was processed using the Empower 2 software (Waters Corp.).

Data analysis, visualization, and statistical analysis

To evaluate synergistic effects of demographic and clinical data a multiple factor analysis was performed with a subsequent permutation test to investigate homogeneity of multivariate dispersion and a permutational multivariate analysis of variance (PERMANOVA) to investigate significant differences between centroids.

For alpha and beta diversity analyses microbiota sequences were rarified to even sequencing depth of 33545 reads per sample. A multiple linear regression model was applied with different demographic and clinical data, and fecal characteristics as predictor and alpha diversity indices as response variable. Subsequently, a type II ANOVA was performed to evaluate effects of individual variables while accounting for effects of other variables. For beta diversity a linear model was fitted to various distance metrics with demographic and clinical data, and fecal characteristics as predictor variables. Subsequently, a permutation test was applied to investigate homogeneity of multivariate dispersion and a PERMANOVA was applied to investigate the effects of individual variables taking effects of other variables into account.

Different taxa ratios were investigated with a linear model with $\log_{10}$ transformed ratio as response variable, and demographic and clinical data, and fecal characteristics as predictor variable, and a subsequent type II ANOVA for evaluation of effects of individual variables. Differential abundance testing was performed using Microbiome Multivariable Associations with Linear Models (MaAsLin2)[35]. Raw counts were transformed to relative abundance and a $\log_2$ transformed linear model was applied with demographic and clinical data as fixed effects. Minimal prevalence was set to 0.25, and significant level was set to 0.05.

Fecal metabolites were investigated with MaAsLin2 applying a linear model with $\log_2$ transformed absolute concentrations of fecal bacterial metabolites normalized to fecal dry weight (µmol/g) and demographic and clinical data, and fecal characteristics as fixed effects. Prediction of functional metabolic potential was performed with Phylogenetic Investigation of Communities by Reconstruction of Unobserved States (PICRUSt2) software[36], calculating MetaCyc pathway abundances[37]. Differential abundance of pathways was investigated with MaAsLin2 as described for bacterial abundance. Prediction accuracy was evaluated using the abundance-weighted nearest sequenced taxon index (NSTI) to summarize the extent to which ASVs in a sample are related to reference 16S rRNA genes. As pain severity and duration of symptoms are confounded with FAP-NOS, effects of these variables were analyzed in corresponding separate models including FAP-NOS data sets only.

Questionnaire for <u>control</u> group

Section 1: General information

Age: __ __

Sex: ☐ male ☐ female

Section 2: Exclusion criteria

Abdominal pain	☐ Yes	☐ No
Non regular bowel movements	☐ Yes	☐ No
Intestinal diseases	☐ Yes	☐ No
Lactose intolerance	☐ Yes	☐ No
Celiac disease	☐ Yes	☐ No
Intestinal infection or diarrhea in last 3 months	☐ Yes	☐ No
Psychiatric illnesses (e.g. ADHD, Autism)	☐ Yes	☐ No
Extreme obesity or anorexia (BMI>P97 or <P3)	☐ Yes	☐ No
Diabetes type 1	☐ Yes	☐ No

If one of the questions was answered with «Yes» the participant is NOT suited for the study

Section 3: Gut microbiota baseline factors

Mode of delivery	☐ Caesarian section	☐ Vaginal delivery
Main infant diet in the first 4 month	☐ Breast fed	☐ Formula fed

If breast fed: How long was the infant solely breast fed?

Pret ______________________________

erm birth (before 37. week of pregnancy)	☐ Yes	☐ No

Section 4: Medication

Intake of **pro**biotics in the last 3 months ☐ Yes ☐ No

If «Yes», what kind of probiotics:

☐ Tablets or powders (e.g. Bioflorin, Perenterol) ☐ Functional foods (e.g. Actimel, LC1, Emmi Bifidus)

Intake of **pre**biotics in the last 3 months (e.g. Inulin) ☐ Yes ☐ No

If «Yes», please specify:

____

____

____

Inta ke of anti biotics in the last 6 months ☐ Yes ☐ No

If «Yes», when?

____

Inta ke of antibiotics in the first 3 years of life ☐ Yes ☐ No

If «Yes», in which year of life ☐ 1 ☐ 2 ☐ 3

(more than one choice possible)

If «Yes», how often ☐ 1-3 ☐ 4-7 ☐ >7

Comments:

____

____

____

Intake of other medication in the last 3 month ☐ Yes ☐ No

If «Yes», what kind of medication:

☐ Imodium ☐ PPI (Proton-pump inhibitor) ☐ PEG (Polyethylene glycol)

☐ Others, please specify:

____

____

____

Section 5: Nutrition

Were dietary preferences changed at any time (were specific diets tested)? ☐ Yes ☐ No

How was the diet changed:

☐ Lactose free diet ☐ Gluten free diet ☐ low FODMAP

☐ Vegan ☐ Vegetarian

☐ Others, please specify:

____

____

____

Start and end of diet change?

____

n the last 6 month ☐ Yes ☐ No

Who informed you about the diet?

☐ Physician ☐ Nutritionist ☐ Self-study (literature, internet)

Consumption of alcohol ☐ Yes ☐ No

If «Yes», since when?

____

Consumption of cigarettes ☐ Yes ☐ No

If «Yes», since when?

____

Questionnaire for <u>IBS/FAP-NOS</u> group

Section 1: General information

Age: __ __

Sex: ☐ male ☐ female

Section 2: Exclusion criteria

Psychiatric illnesses (e.g. ADHD, Autism)	☐ Yes	☐ No
Extreme obesity or anorexia (BMI>P97 or <P3)	☐ Yes	☐ No
Diabetes type 1	☐ Yes	☐ No

If one of the questions was answered with «Yes» the participant is NOT suited for the study

Section 3: Diagnosis

Diagnosis ☐ IBS ☐ FAP-NOS

For IBS:

Which subgroup ☐ IBS-D ☐ IBS-C ☐ IBS-M ☐ IBS-U

Did the symptoms start after an intestinal infection or diarrhea ☐ Yes ☐ No

Pain intensity ☐ 1 ☐ 2 ☐ 3 ☐ 4 ☐ 5 ☐ 6 ☐ 7 ☐ 8 ☐ 9 ☐ 10
(Answer this question with the pain chart)

How long are the symptoms present __ __ months

Section 4: Gut microbiota baseline factors

Mode of delivery ☐ Caesarian section ☐ Vaginal delivery

Main infant diet in the first 4 month ☐ Breast fed ☐ Formula fed

If breast fed: How long was the infant solely breast fed?

Pret

erm birth ☐ Yes ☐ No
(before 37. week of pregnancy)

Section 5: Medication

Intake of **pro**biotics in the last 3 months ☐ Yes ☐ No

If «Yes», what kind of probiotics:

☐ Tablets or powders (e.g. Bioflorin, Perenterol) ☐ Functional foods (e.g. Actimel, LC1, Emmi Bifidus)

Intake of **pre**biotics in the last 3 months (e.g. Inulin) ☐ Yes ☐ No

If «Yes», please specify:

____

____

____

Intake of antibiotics in the last 6 months ☐ Yes ☐ No

If «Yes», when?

____

Intake of antibiotics in the first 3 years of life ☐ Yes ☐ No

If «Yes», in which year of life ☐ 1 ☐ 2 ☐ 3
(more than one choice possible)

If «Yes», how often ☐ 1-3 ☐ 4-7 ☐ >7

Comments:

____

____

____

Intake of other medication in the last 3 month ☐ Yes ☐ No

If «Yes», what kind of medication:

☐ Imodium ☐ PPI (Proton-pump inhibitor) ☐ PEG (Polyethylene glycol)

☐ Others, please specify:

____

____

____

Section 6: Nutrition

Were dietary preferences changed at any time (were specific diets tested)? ☐ Yes ☐ No

How was the diet changed:

☐ Lactose free diet ☐ Gluten free diet ☐ low FODMAP

☐ Vegan ☐ Vegetarian

☐ Others, please specify:

___

___

___

Start and end of diet change?

___

n the last 6 month ☐ Yes ☐ No

Who informed you about the diet?

☐ Physician ☐ Nutritionist ☐ Self-study (literature, internet)

Consumption of alcohol ☐ Yes ☐ No

If «Yes», since when?

___

Consumption of cigarettes ☐ Yes ☐ No

If «Yes», since when?

___

Supplementary Figures

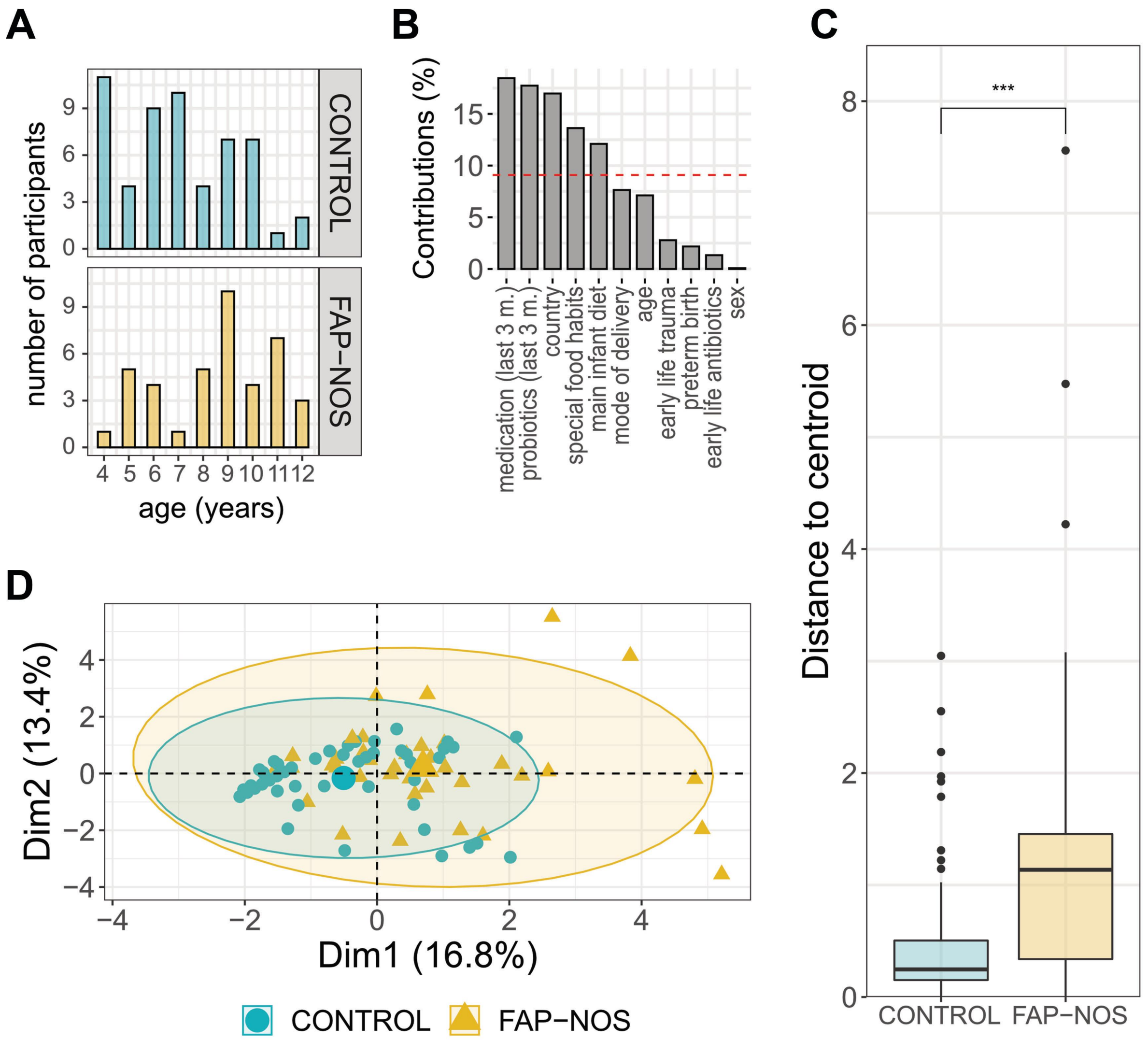

Supplementary Figure 1. Mixed data factor analysis of demographic and clinical data of FAP-NOS and control groups. (A) Age distribution for control and FAP-NOS groups; (D) Visualization of mixed factor analysis comparing control (blue circles) and FAP-NOS (orange triangles) groups. The small symbols display individuals, the large symbols display centroids, and ellipses indicate 95% of confidence intervals; (C) Differences in multivariate dispersion between control and FAP-NOS groups, visualized as distance to centroid. Boxplot with box elements showing upper and lower quantile and median. Whiskers extend from the upper/lower quantile to ± 1.5 iqr or the highest/lowest value. Outliers are indicated as black points. Significance in multivariant dispersion was calculated with permutation test; (B) All variables included in mixed factor analysis contributing to variability across Dim1 axis in descending order. Red reference dashed line indicates expected value if contributions were uniform. ***$P < 0.001$; m.: month

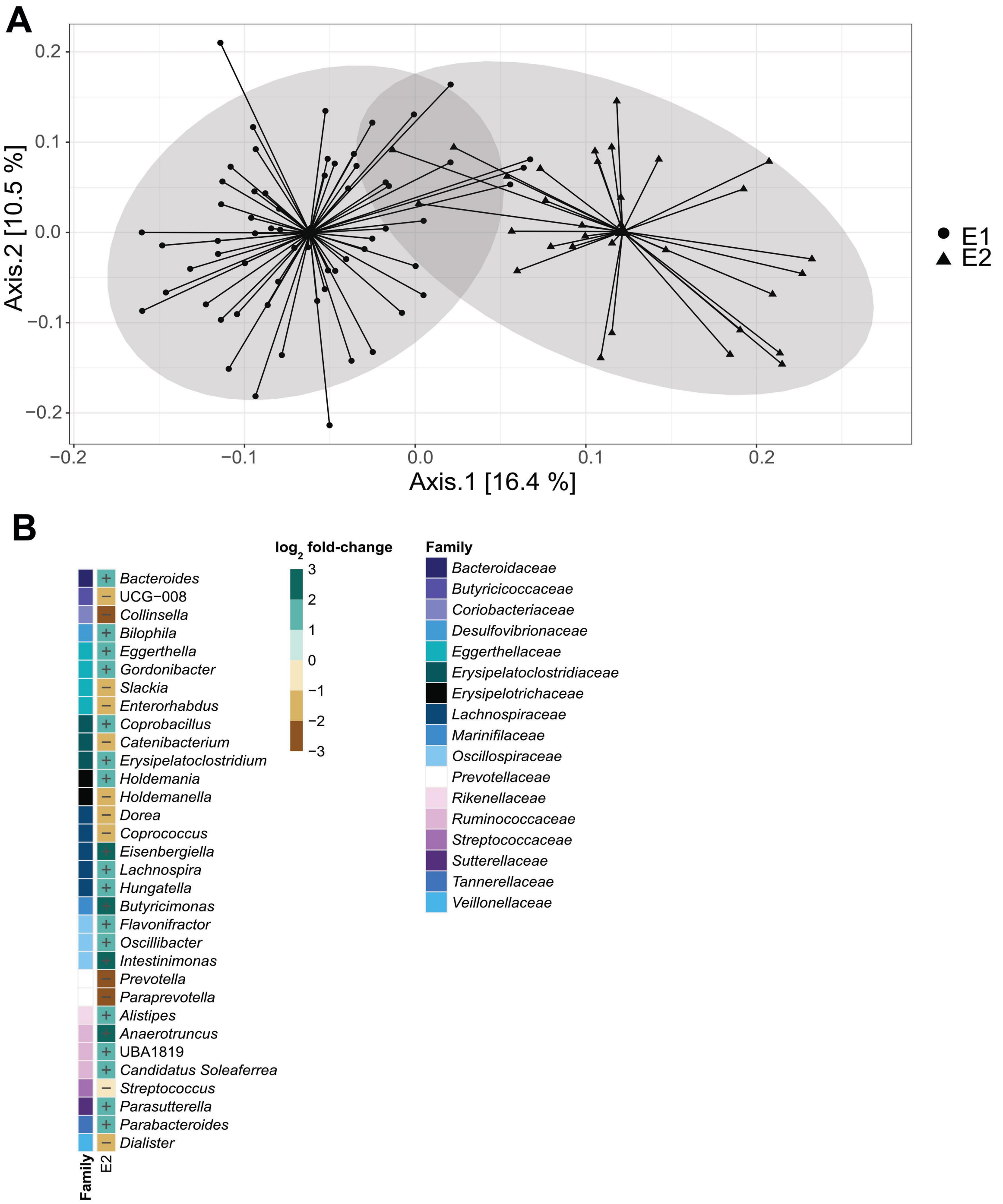

Supplementary Figure 2. Characterization of *de novo* enterotypes. (A) Clustering of total study participant microbiota (n = 95) at genus level based on Jensen-Shannon divergence and partitioning around medoids (PAM) clustering algorithm. Microbiota assigned to enterotype 1 (E1) are depicted as circles, microbiota assigned to enterotype 2 (E2) are depicted as triangles, and ellipses indicate 95 % of confidence intervals; (B) Log_2 fold-change of significant ($P < 0.05$) differentially abundant genera in E2 compared to E1. Additional taxonomic information is indicated at family level. Significances were calculated using a log_2-transformed linear model with *de novo* enterotype as predictor and abundances as response variable and FDR correction for multiple testing.

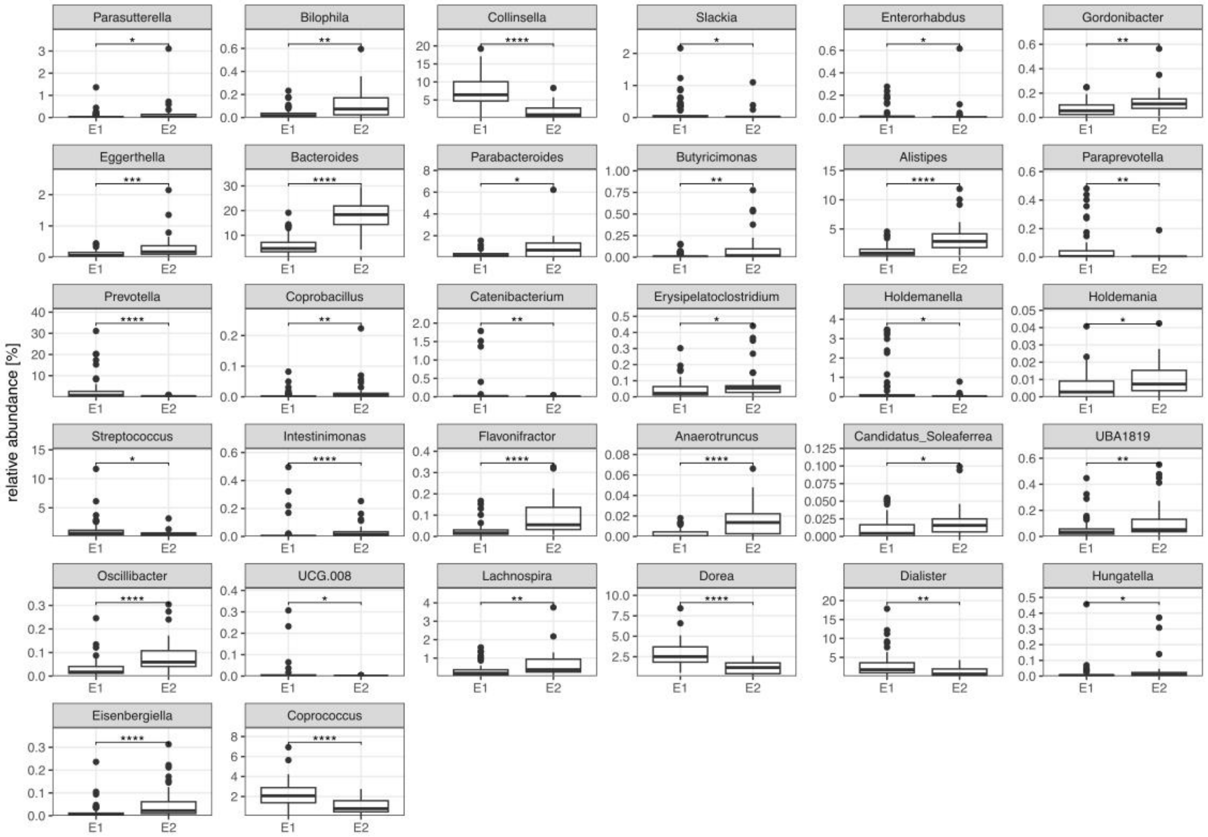

Supplementary Figure 3. Differential abundance of genera in enterotype 1 and 2. Relative abundance of significantly ($P < 0.05$) increased or decreased genera in enterotype 1 when compared with enterotype 2. Boxplot with box elements showing upper and lower quantile and median. Whiskers extend from the upper/lower quantile to ± 1.5 iqr or the highest/lowest value. Outliers are indicated as black points. Significances were calculated using a log_2-transformed linear model with *de novo* enterotype as predictor and abundances as response variable and FDR correction for multiple testing.

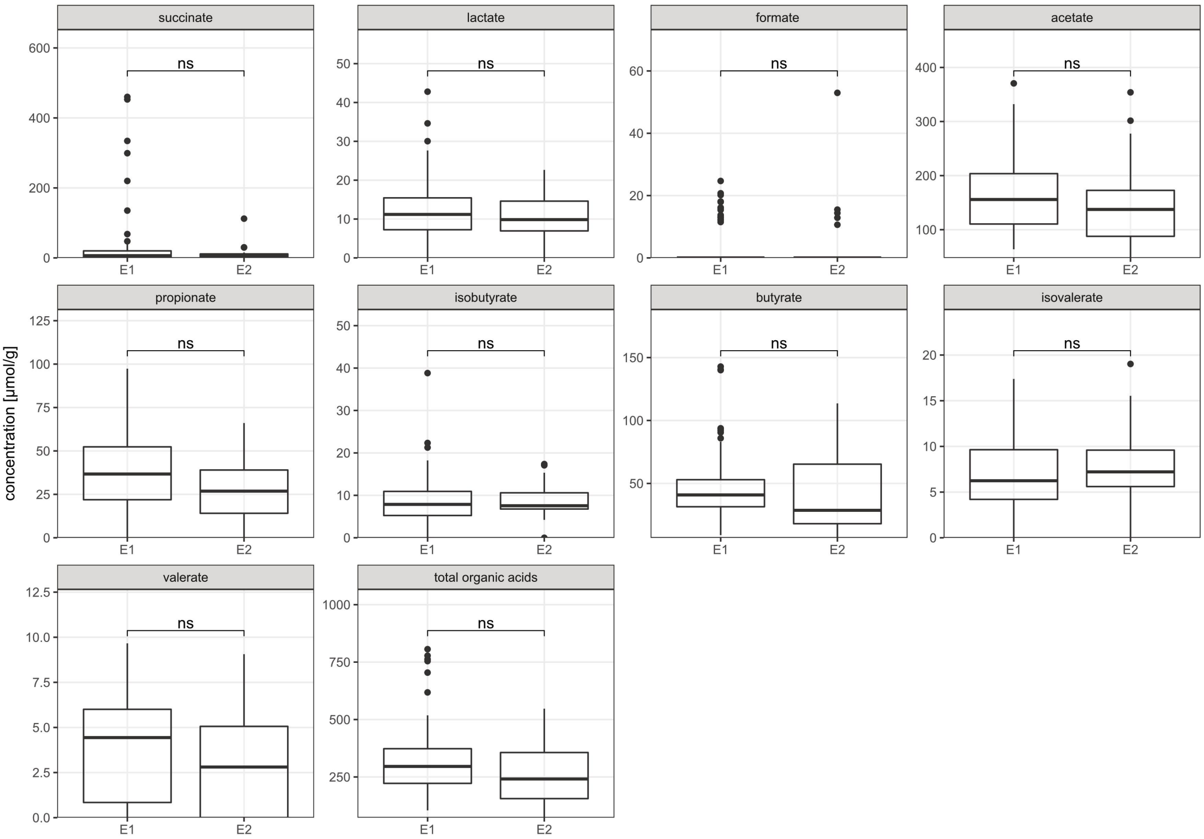

Supplementary Figure 4. Comparison of total and specific organic acid concentrations between enterotype 1 (E1) and enterotype 2 (E2) microbiota. Metabolite concentration is depicted per gram fecal dry weight. Boxplot with box elements showing upper and lower quantile and median. Whiskers extend from the upper/lower quantile to ± 1.5 iqr or the highest/lowest value. Outliers are indicated as black points. Significances were calculated using Wilcoxon rank-sum test including FDR correction. ns: not significant.

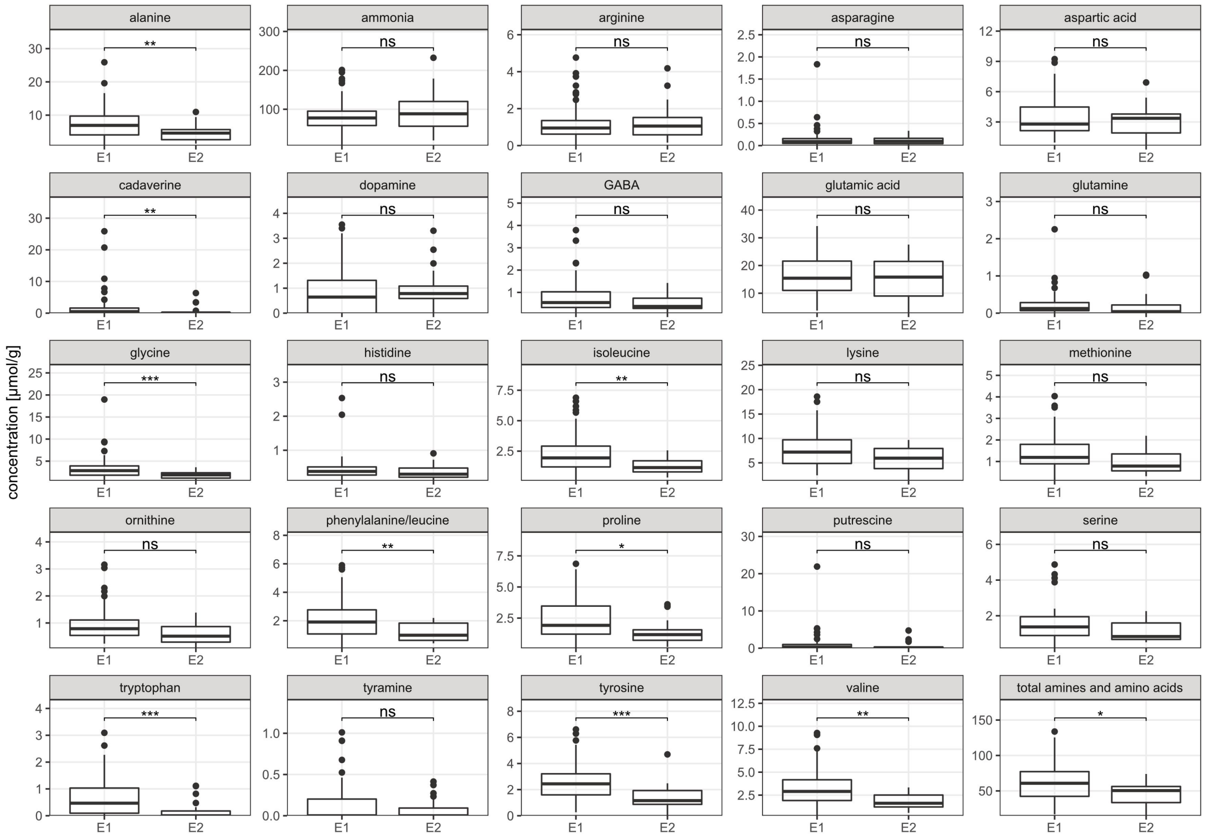

Supplementary Figure 5: Comparison of amine, amino acid, and ammonia concentrations between enterotype 1 (E1) and enterotype 2 (E2) microbiota. Metabolite concentration is depicted per gram fecal dry weight. Boxplot with box elements showing upper and lower quantile and median. Whiskers extend from the upper/lower quantile to ± 1.5 iqr or the highest/lowest value. Outliers are indicated as black points. Concentrations of phenylethylamine were under the detection limit. Significances were calculated using Wilcoxon rank-sum test including FDR correction. $*P < 0.05$; $**P < 0.01$;$***P < 0.001$; ns: not significant; GABA: γ-aminobutyric acid.

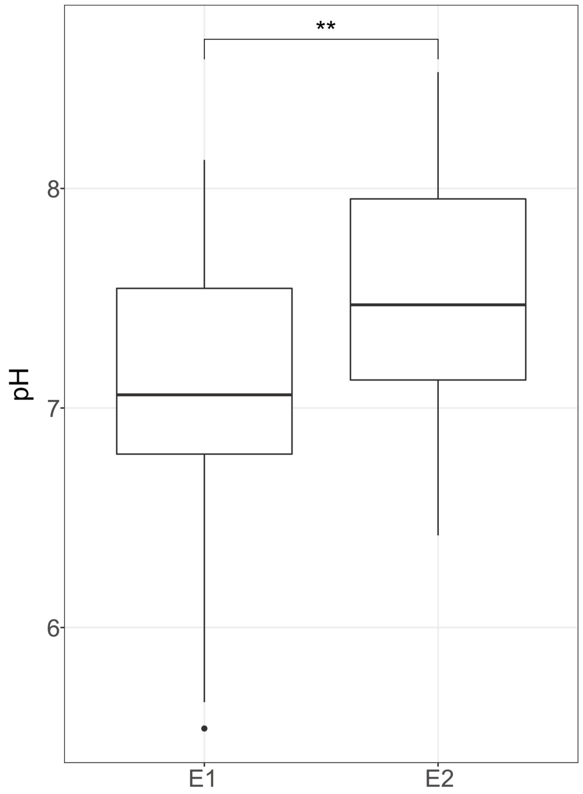

Supplementary Figure 6. Comparison of fecal pH between enterotype 1 (E1) and enterotype 2 (E2) microbiota. Boxplot with box elements showing upper and lower quantile and median. Whiskers extend from the upper/lower quantile to ± 1.5 iqr or the highest/lowest value. Outliers are indicated as black points. Significances were calculated using Wilcoxon rank-sum test. $**P < 0.01$.

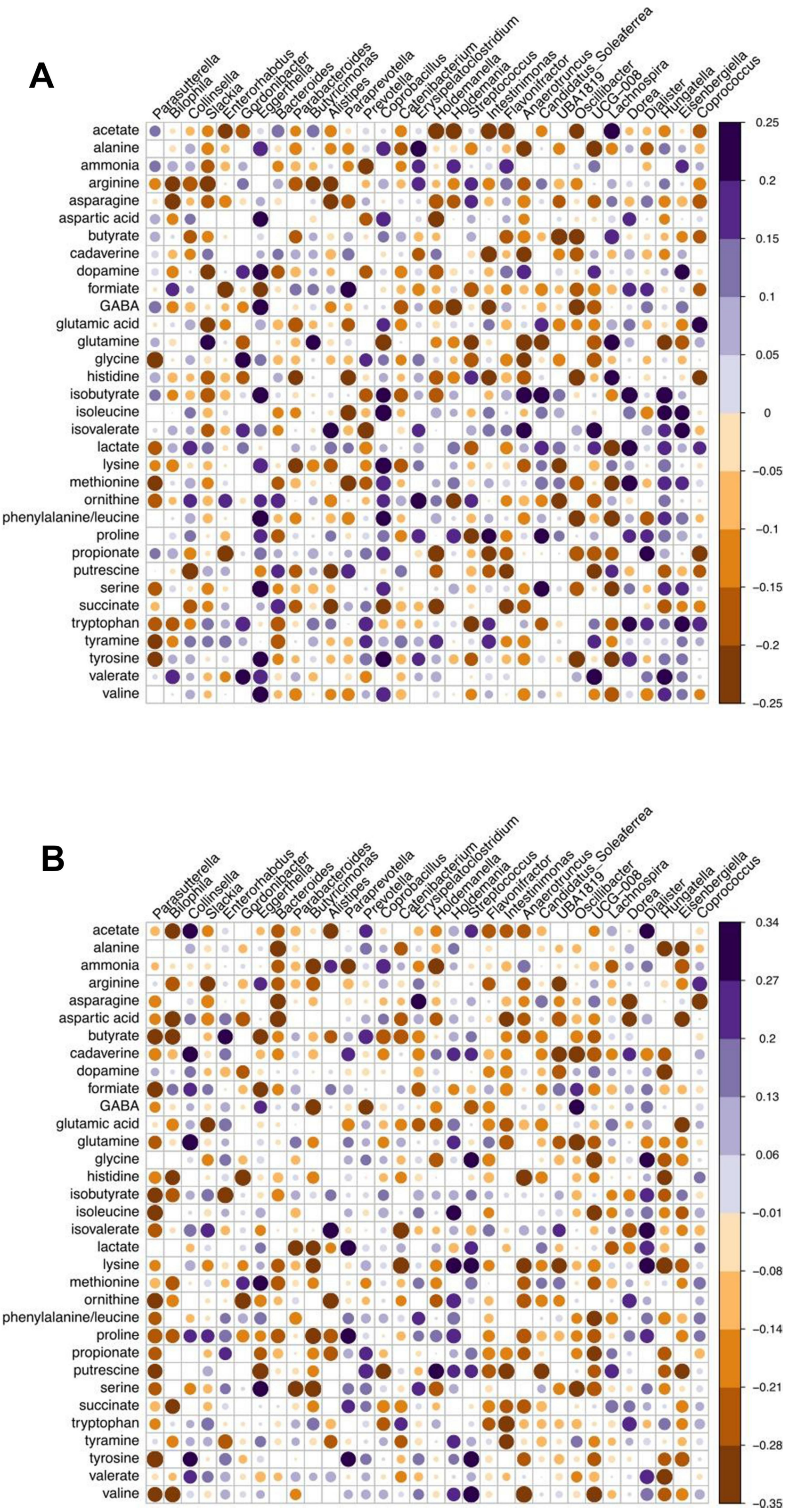

Supplementary Figure 7. Correlation between significantly differentially abundant genera in enterotype 1 and 2, and fecal metabolites. Spearman correlation matrix of significantly differentially abundant genera and metabolites for (A) enterotype1 and (B) enterotype 2. Significant ($P < 0.05$). correlations are indicated as dots. Positive correlation coefficients (blue) and negative correlation coefficients (red)**.**

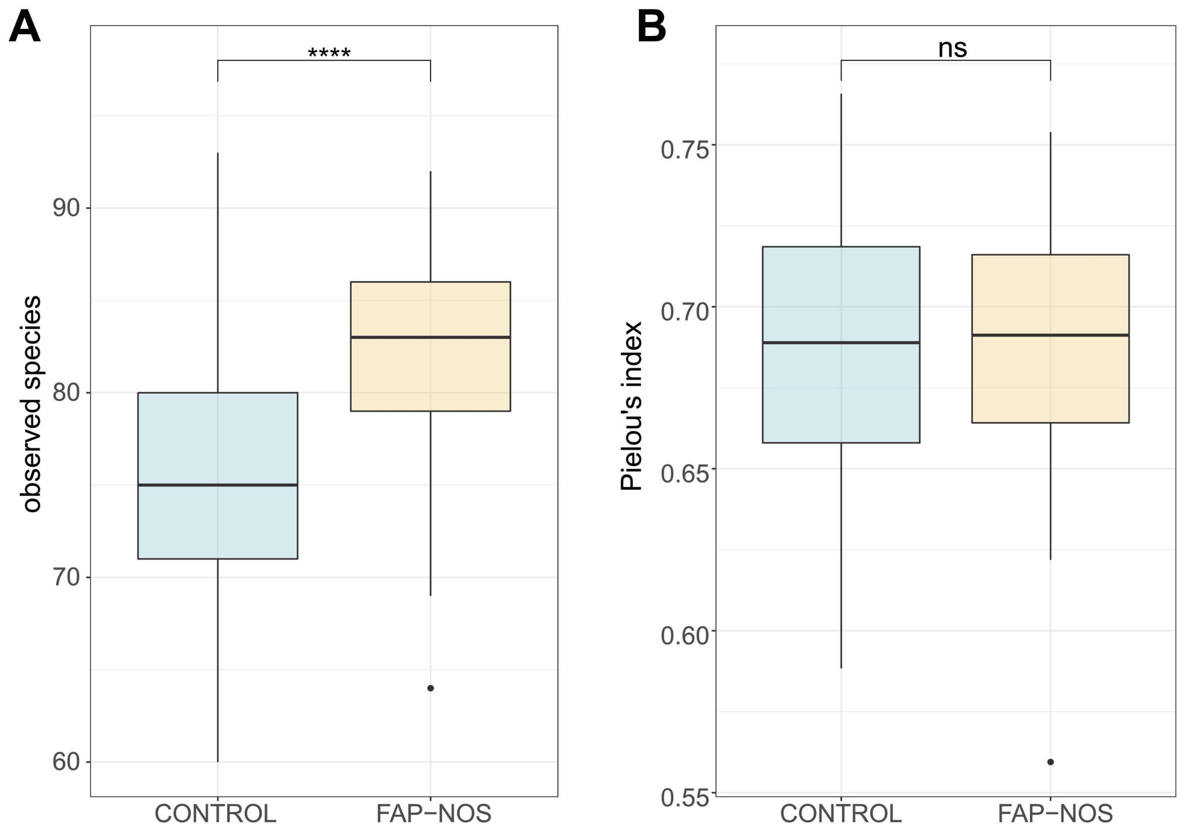

Supplementary Figure 8. Alpha diversity indices at species level. Comparison of (A) richness and (B) evenness between FAP-NOS and control groups. Boxplot with box elements showing upper and lower quantile and median. Whiskers extend from the upper/lower quantile to ± 1.5 iqr or the highest/lowest value. Outliers are indicated as black points. All significances were calculated using a multiple linear regression model [Supplementary Table 4]. ns: not significant; ****$P < 0.0001$.

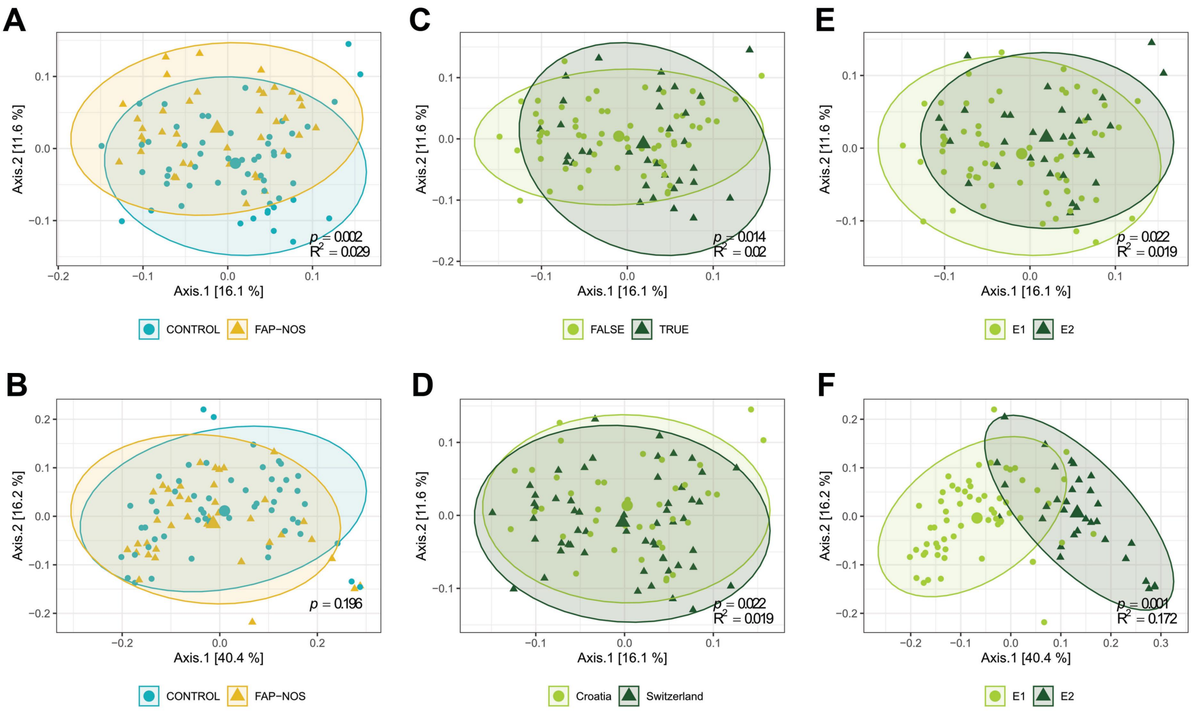

Supplementary Figure 9. Beta diversity metrics at species level. Comparison of (A) unweighted and (B) weighted Unifrac between FAP-NOS and control groups; Visualization of effect of (C) probiotics treatment in the last three months (FALSE *vs.* TRUE), (D) country, and (E) *de novo* enterotype on unweighted Unifrac; (F) Visualization of effect of enterotype on weighted Unifrac. Small data points display individual microbiota, large data points display centroids, and ellipses indicate 95% of confidence intervals. All significances were calculated using a linear model fitted to various distance metrices [Supplementary Tables 7 and 8].

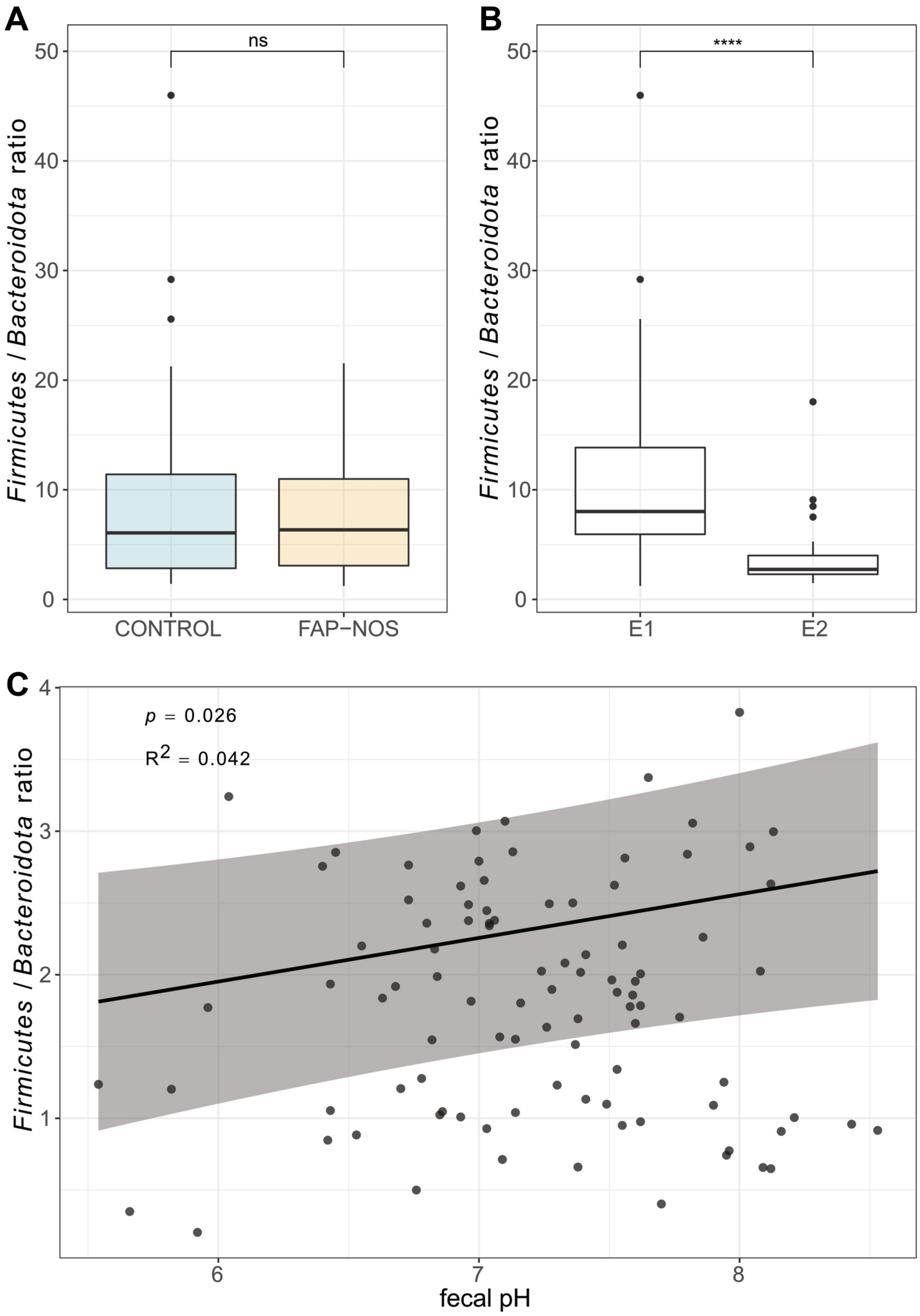

Supplementary Figure 10. *Firmicutes/Bacteroidetes* ratio. Comparison of *Firmicutes/Bacteroidetes* ratio (A) between control and FAP-NOS groups and between (B) *de novo* enterotypes. Boxplot with box elements showing upper and lower quantile and median. Whiskers extend from the upper/lower quantile to ± 1.5 iqr or the highest/lowest value. Outliers are indicated as black points; (C) Correlation of *Firmicutes/Bacteroidetes* ratio with fecal pH. Points in scatterplot display values for individual microbiota. Regression line is based on multiple linear regression model, and 95% confidence interval is displayed. All significances were calculated using a multiple linear regression model [Supplementary Table 9]. $****P < 0.0001$; ns: not significant.

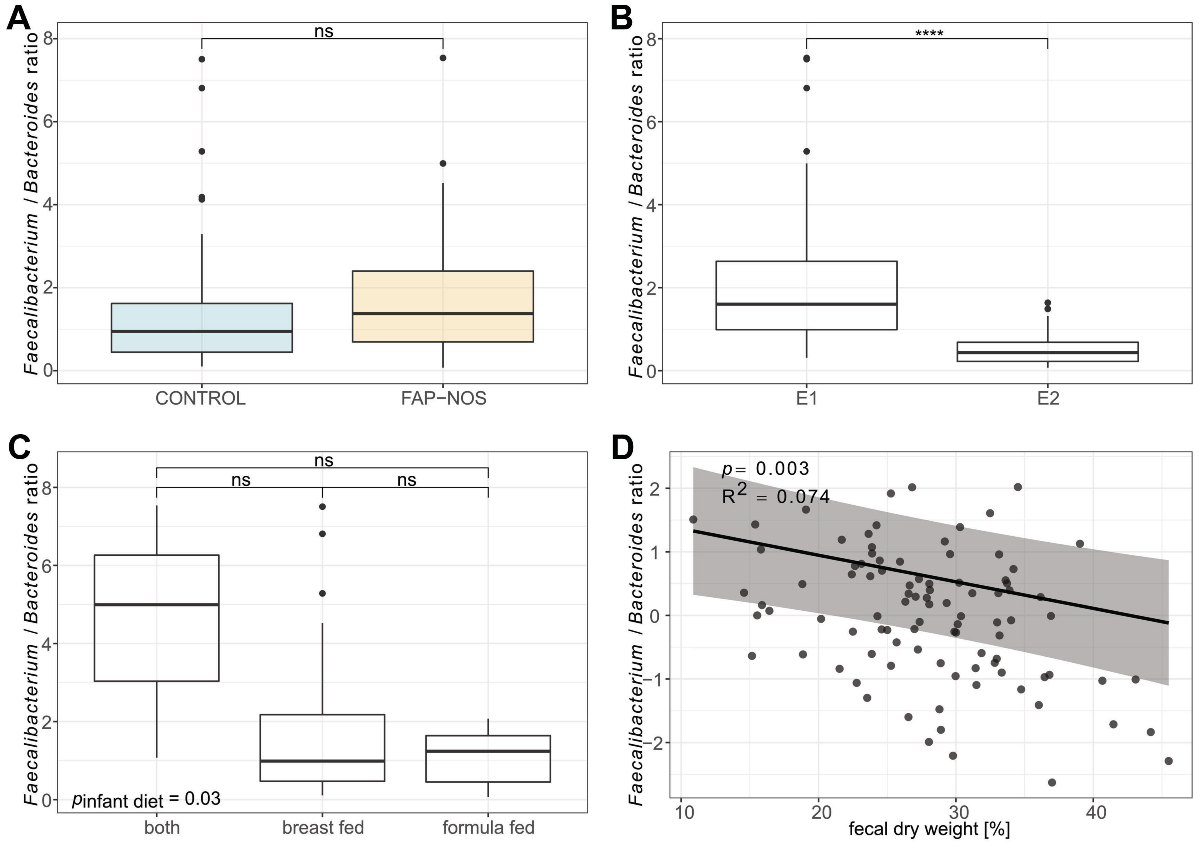

Supplementary Figure 11. *Faecalibacterium*/*Bacteroides* ratio. Comparison of *Faecalibacterium*/*Bacteroides* ratio (A) between control and FAP-NOS groups, between (B) *de novo* enterotypes, and between (C) different infant diets. For infant diets overall significance ($p_{\text{infant diet}}$) is indicated in the graph. Boxplot with box elements showing upper and lower quantile and median. Whiskers extend from the upper/lower quantile to ± 1.5 iqr or the highest/lowest value. Outliers are indicated as black points; (D) Correlation of *Firmicutes*/*Bacteroidetes* ratio with fecal dry weight. Points in scatterplot display values for individual microbiota. Regression line is based on multiple linear regression model, and 95% confidence interval is displayed. All significances were calculated using a multiple linear regression model [Supplementary Table 10]. *****P* < 0.0001; ns: not significant.

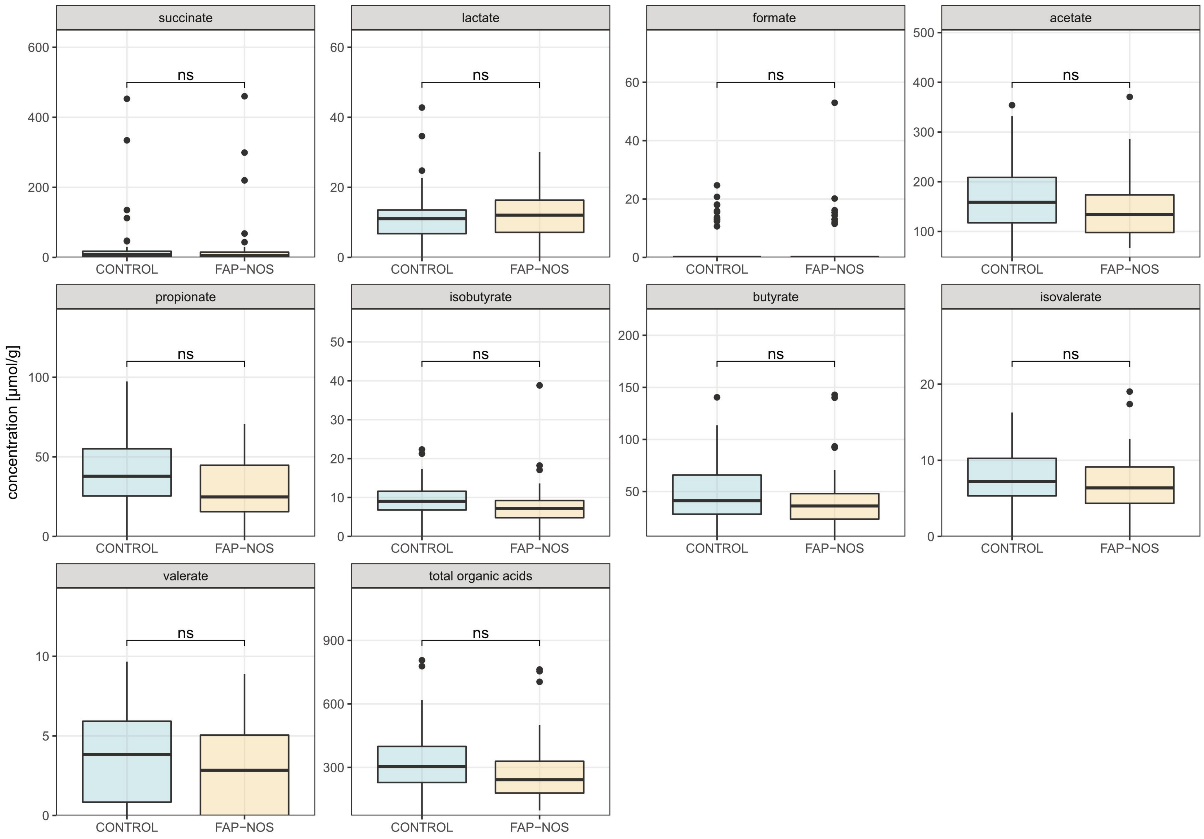

Supplementary Figure 12. Comparison of fecal specific and total organic acid concentrations between FAP-NOS and controls. Metabolite concentration is depicted per gram fecal dry weight. Boxplot with box elements showing upper and lower quantile and median. Whiskers extend from the upper/lower quantile to ± 1.5 iqr or the highest/lowest value. Outliers are indicated as black points. Significances were calculated using linear models with FDR correction for multiple testing. ns: not significant.

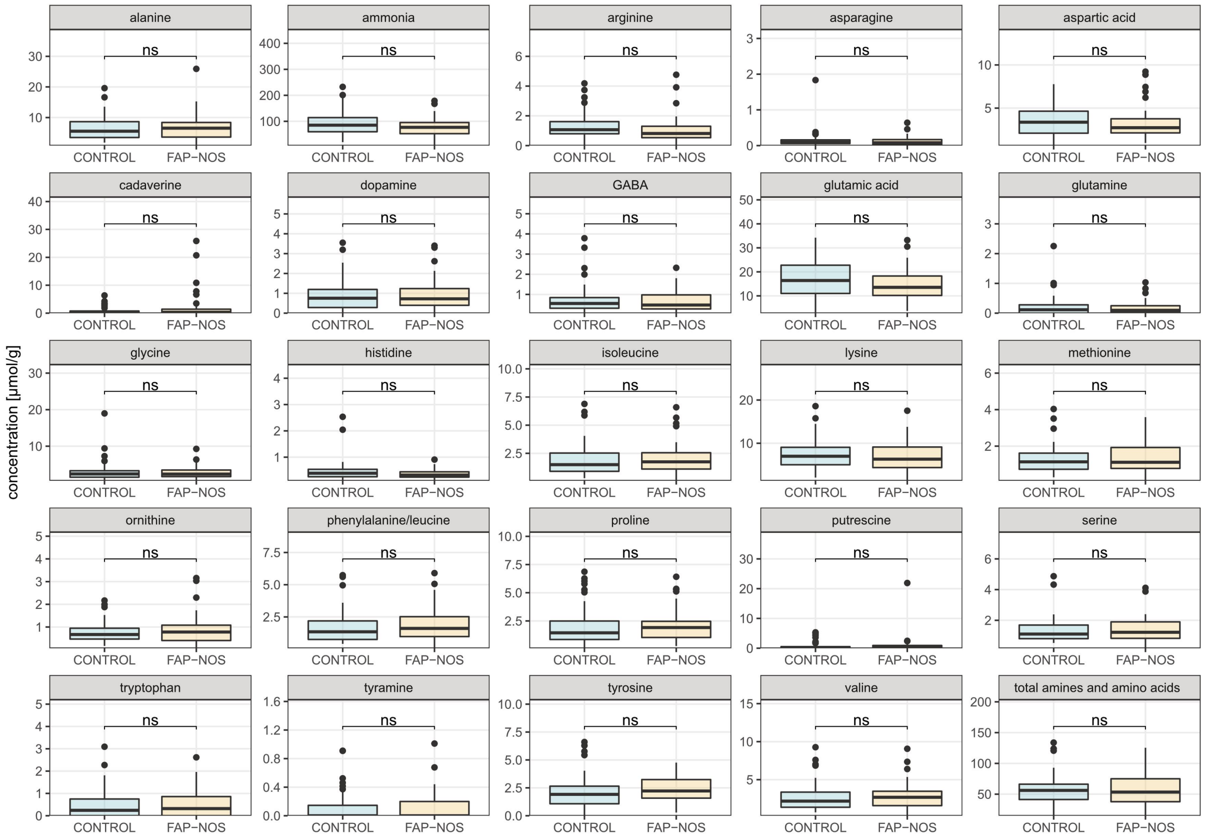

Supplementary Figure 13. Comparison of amine, amino acid, and ammonia concentrations between FAP-NOS and controls. Metabolite concentration is depicted per gram fecal dry weight. Boxplot with box elements showing upper and lower quantile and median. Whiskers extend from the upper/lower quantile to ± 1.5 iqr or the highest/lowest value. Outliers are indicated as black points. Concentrations of phenylethylamine were under the detection limit. Significances were calculated using linear models with FDR correction for multiple testing. ns: not significant. GABA: γ-aminobutyric acid

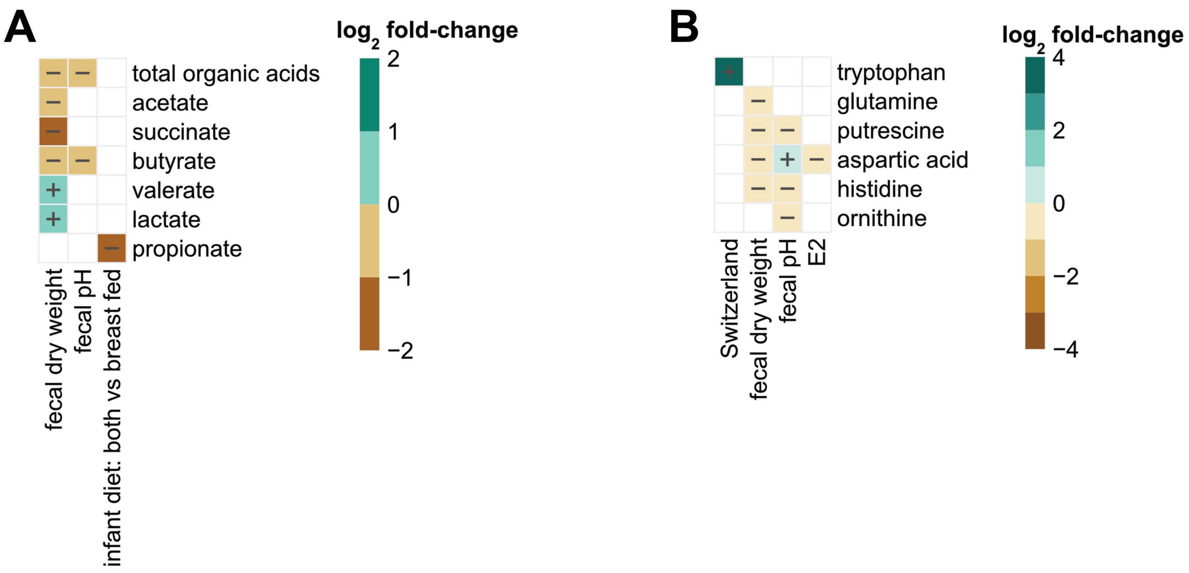

Supplementary Figure 14. Fecal (A) organic acids, (B) amino acids and amines with significantly (*P* < 0.05) different concentrations in demographic and clinical data, and fecal characteristics. Log_2-fold-change in fecal metabolites per gram fecal dry weight per unit of continuous variables or compared to corresponding categorical variable is displayed. Significances were calculated using linear models with FDR correction for multiple testing.

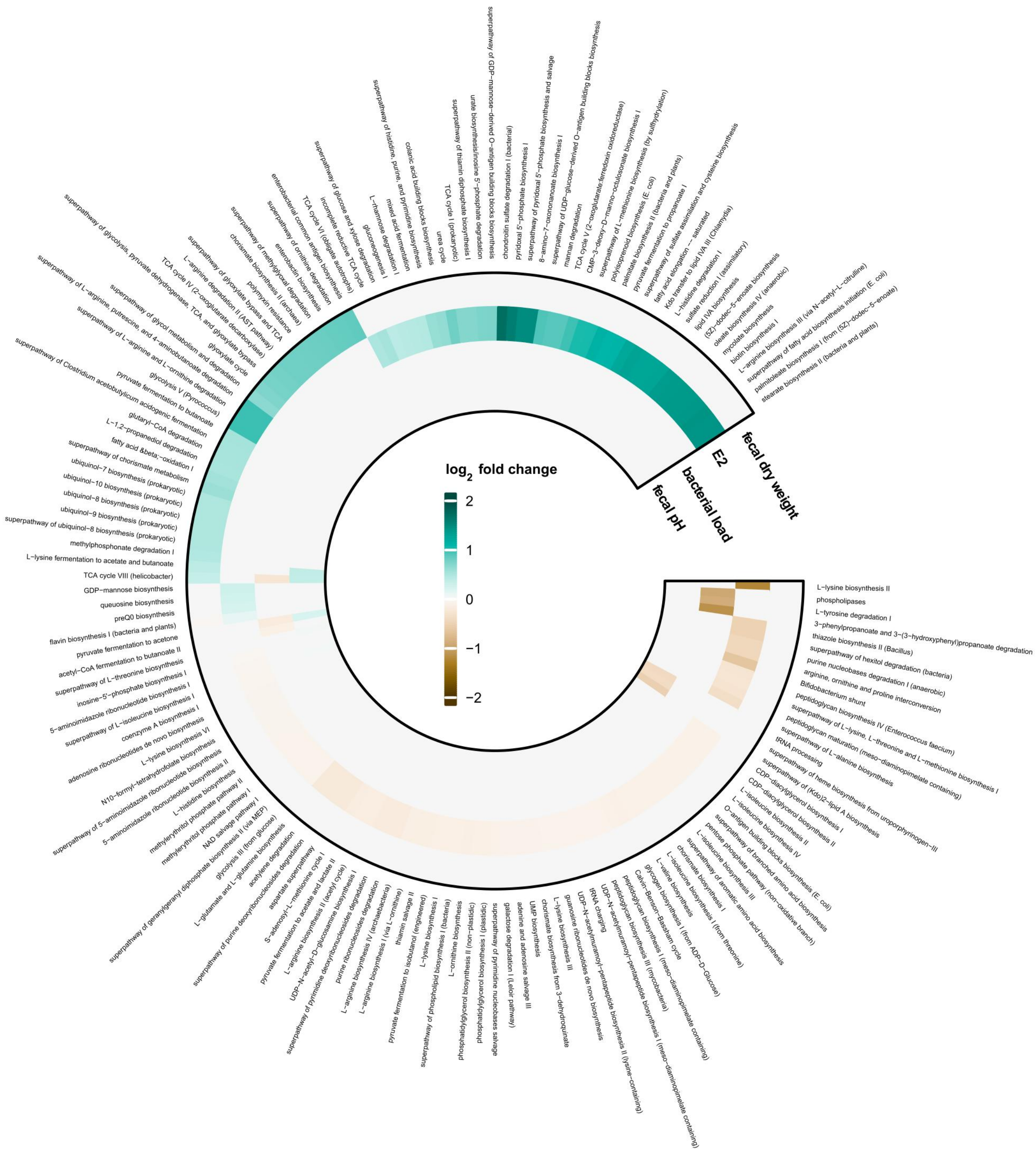

Supplementary Figure 15. Abundance $\log_2$ fold-change of predicted metabolic pathways correlating with or significantly (P < 0.05) different in categories of demographic and clinical data, and fecal characteristics. Log_2 fold-change in abundance of predicted pathways per unit of continuous variables or compared to corresponding categorical variable is displayed. Significances were calculated using $\log_2$ transformed linear models with FDR correction for multiple testing. Bacterial load refers to $\log_{10}$ bacterial total 16S rRNA gene copies per dry weight.

Supplementary Tables:

Supplementary Table 1. General early life events in the first three years of life, assessed by caregivers *via* Life Event Scale modified for early life. Data is given in percentage of total participants per group (healthy controls *vs.* FAP-NOS). Significances were calculated using Fisher's exact test including FDR correction

	**Healthy controls**	**FAP-NOS**	***P*-value***
Pregnancy or birth of caregiver	34.5%	25.0%	ns
Separation or divorce of caregiver	12.7%	10.0%	ns
Marriage/re-marrying of caregiver	5.5%	2.5%	ns
Moving in of relative or another known person	7.3%	7.5%	ns
Substantial change in family income	7.3%	2.5%	ns
Serious debt	0%	0%	na
Relocation/change of residence	29.1%	32.5%	ns
Change of job of caregiver or change of job of spouse	30.9%	30.0%	ns
Unemployment of caregiver or unemployment of the spouse	9.1%	17.5%	ns
Serious sickness or accident of a family member	16.4%	7.5%	ns
Death in the family or in the close circle of friends	27.3%	5.0%	ns
Start of child going to daycare center	78.2%	60.0%	ns
Change of daycare center	21.8%	10.0%	ns
Sleep disorder of child	12.7%	22.5%	ns

*FDR-adjusted *p*-values; ns: not significant; na: not applicable.

Supplementary Table 2. Early life trauma in the first three years of life, assessed by YCPC or modified UCLA PTSD Reaction Index for DSM-IV caregiver reports, aggregated to five different categories. Data is given in percentage of total participants per group (healthy controls *vs.* FAP-NOS). Significances were calculated using Fisher's exact test including FDR correction

	**Healthy controls**	**FAP-NOS**	***P*-value***
Experiencing natural disasters, severe accidents, and/or war	0%	2.5%	ns
Experiencing physical and/or sexual abuse	0%	0%	na
Witnessing of physical abuse	1.8%	0%	ns
Experiencing stressful medical treatment	9.1%	7.5%	ns
Experiencing other traumatic events	1.8%	5%	ns

*FDR-adjusted p-values; ns: not significant; na: not applicable.

Supplementary Table 3. ANOVA table of multiple linear regression model used to explore gut microbial richness at ASV level

Variable	Sum Sq	Df	F value	*P*-value	R^2
Control *vs.* FAP-NOS	49,724.50	1	39.60	$1.90*10^{-08}$	0.27
Age	888.41	1	0.71	0.40	$4.84*10^{-03}$
Sex	2,419.13	1	1.93	0.17	0.01
Country	4,212.59	1	3.35	0.07	0.02
Mode of delivery	660.81	1	0.53	0.47	$3.60*10^{-03}$
Main infant diet	4,116.38	2	1.64	0.20	0.02
Preterm birth	1,156.49	1	0.92	0.34	0.01
Early life traumatic events	32.90	1	0.03	0.87	$1.79*10^{-04}$
Early life antibiotics treatment	550.16	1	0.44	0.51	$3.00*10^{-03}$
Probiotics treatment in the last three months	5,241.68	1	4.17	0.04	0.03
Lactose specific diet	3,920.55	2	1.56	0.22	0.02
PEG	153.33	1	0.12	0.73	$8.36*10^{-04}$
PPI	16.13	1	0.01	0.91	$8.80*10^{-05}$
Fecal dry weight	5,250.12	1	4.18	0.04	0.03
Log_{10} 16S rRNA gene copies per g dry weight	685.06	1	0.55	0.46	$3.74*10^{-03}$
De novo enterotype	2,581.62	1	2.06	0.16	0.01
Fecal pH	7,588.56	1	6.04	0.02	0.04
Residuals	94,183.91	75	NA	NA	0.51

Df: degrees of freedom; PEG: polyethylene glycol; PPI: proton pump inhibitors; Sum Sq: sum of squares.

Supplementary Table 4. ANOVA table of multiple linear regression model used to explore gut microbial richness at species level

Variable	Sum Sq	Df	F value	*P*-value	R^2
Control *vs.* FAP-NOS	848.57	1	19.53	$3.29*10^{-05}$	0.17
Age	31.69	1	0.73	0.40	0.01
Sex	16.79	1	0.39	0.54	$3.45*10^{-03}$
Country	0.46	1	0.01	0.92	$9.51*10^{-05}$
Mode of delivery	10.11	1	0.23	0.63	$2.08*10^{-03}$
Main infant diet	140.13	2	1.61	0.21	0.03
Preterm birth	35.25	1	0.81	0.37	0.01
Early life traumatic events	2.57	1	0.06	0.81	$5.28*10^{-04}$
Early life antibiotics treatment	6.96	1	0.16	0.69	$1.43*10^{-03}$
Probiotics treatment in the last three months	129.37	1	2.98	0.09	0.03
Lactose specific diet	141.13	2	1.62	0.20	0.03
PEG	48.06	1	1.11	0.30	0.01
PPI	7.41	1	0.17	0.68	$1.52*10^{-03}$
Fecal dry weight	130.72	1	3.01	0.09	0.03
Log_{10} 16S rRNA gene copies per g dry weight	29.70	1	0.68	0.41	0.01
De novo enterotype	4.34	1	0.10	0.75	$8.93*10^{-04}$
Fecal pH	22.35	1	0.51	0.48	$4.59*10^{-03}$
Residuals	3,258.46	75	NA	NA	0.67

Df: degrees of freedom; NA: not applicable; PEG: polyethylene glycol; PPI: proton pump inhibitors; Sum Sq: sum of squares.

Supplementary Table 5. PERMANOVA table of linear model fitted to unweighted Unifrac distance metric at ASV level, including analysis of multivariate homogeneity of group dispersions

Variable	Sum Sq	Df	F value	*P*-value	R^2	Dispersion *P*-value*
Control *vs.* FAP-NOS	0.14	1	2.05	$1.00*10^{-03}$	0.02	0.09
Age	0.09	1	1.37	0.06	0.01	NA
Sex	0.07	1	1.02	0.41	0.01	0.15
Country	0.14	1	2.05	$1.00*10^{-03}$	0.02	0.09
Mode of delivery	0.05	1	0.80	0.85	0.01	0.40
Main infant diet	0.12	2	0.89	0.78	0.02	0.04
Preterm birth	0.06	1	0.84	0.83	0.01	0.15
Early life traumatic events	0.06	1	0.82	0.83	0.01	0.09
Early life antibiotics treatment	0.07	1	1.02	0.43	0.01	0.25
Probiotics treatment in the last three months	0.09	1	1.32	0.08	0.01	0.09
Lactose specific diet	0.12	2	0.89	0.77	0.02	0.01
PEG	0.05	1	0.69	0.97	0.01	0.02
PPI	0.05	1	0.77	0.91	0.01	0.01
Fecal dry weight	0.13	1	1.97	$1.00*10^{-03}$	0.02	NA
Log_{10} 16S rRNA gene copies per g dry weight	0.09	1	1.28	0.08	0.01	NA
De novo enterotype	0.12	1	1.81	$2.00*10^{-03}$	0.02	0.15
Fecal pH	0.10	1	1.38	0.04	0.01	NA
Residuals	5.13	75	NA	NA	0.74	NA

*FDR-adjusted; Df: degrees of freedom; NA: not applicable; PEG: polyethylene glycol; PPI: proton pump inhibitors; Sum Sq: sum of squares.

Supplementary Table 6. PERMANOVA table of linear model fitted to weighted Unifrac distance metric at ASV level, including analysis of multivariate homogeneity of group dispersions

Variable	Sum Sq	Df	F value	*P*-value	R^2	dispersion *P*-value*
Control *vs.* FAP-NOS	0.02	1	0.69	0.67	0.01	0.88
Age	0.02	1	0.90	0.50	0.01	NA
Sex	0.02	1	0.91	0.45	0.01	0.97
Country	0.02	1	0.92	0.44	0.01	0.35
Mode of delivery	0.01	1	0.51	0.85	$4.00*10^{-03}$	0.97
Main infant diet	0.04	2	0.94	0.47	0.02	0.35
Preterm birth	0.01	1	0.64	0.70	0.01	0.97
Early life traumatic events	0.01	1	0.37	0.94	$3.00*10^{-03}$	0.35
Early life antibiotics treatment	0.02	1	0.78	0.58	0.01	0.87
Probiotics treatment in the last three months	0.02	1	0.73	0.62	0.01	0.35
Lactose specific diet	0.03	2	0.64	0.84	0.01	0.40
PEG	0.03	1	1.17	0.30	0.01	0.97
PPI	0.01	1	0.64	0.72	0.01	0.57
Fecal dry weight	0.04	1	1.61	0.12	0.01	NA
Log_{10} 16S rRNA gene copies per g dry weight	0.05	1	2.18	0.04	0.02	NA
De novo enterotype	0.35	1	15.73	$1.00*10^{-03}$	0.14	0.97
Fecal pH	0.05	1	2.09	0.04	0.02	NA
Residuals	1.67	75	NA	NA	0.64	NA

*FDR-adjusted; Df: degrees of freedom; NA: not applicable; PEG: polyethylene glycol; PPI: proton pump inhibitors; Sum Sq: sum of squares.

Supplementary Table 7. PERMANOVA table of linear model fitted to unweighted Unifrac distance metric at species level, including analysis of multivariate homogeneity of group dispersions

Variable	Sum Sq	Df	F value	*P*-value	R^2	dispersion *P*-value*
Control *vs.* FAP-NOS	0.09	1	2.93	$2.00*10^{-03}$	0.03	0.32
Age	0.02	1	0.77	0.73	0.01	NA
Sex	0.03	1	1.15	0.29	0.01	0.66
Country	0.06	1	1.93	0.02	0.02	0.38
Mode of delivery	0.02	1	0.58	0.90	0.01	0.66
Main infant diet	0.05	2	0.93	0.60	0.02	0.47
Preterm birth	0.05	1	1.56	0.07	0.02	0.47
Early life traumatic events	0.02	1	0.56	0.91	0.01	0.66
Early life antibiotics treatment	0.02	1	0.76	0.72	0.01	0.66
Probiotics treatment in the last three months	0.06	1	2.03	0.01	0.02	0.66
Lactose specific diet	0.04	2	0.74	0.84	0.02	0.32
PEG	0.02	1	0.82	0.67	0.01	0.38
PPI	0.03	1	1.03	0.42	0.01	0.03
Fecal dry weight	0.04	1	1.47	0.10	0.02	NA
Log_{10} 16S rRNA gene copies per g dry weight	0.02	1	0.73	0.74	0.01	NA
De novo enterotype	0.06	1	1.92	0.02	0.02	0.67
Fecal pH	0.03	1	0.99	0.46	0.01	NA
Residuals	2.19	75	NA	NA	0.74	NA

*FDR-adjusted; Df: degrees of freedom; NA: not applicable; PEG: polyethylene glycol; PPI: proton pump inhibitors; Sum Sq: sum of squares.

Supplementary Table 8. PERMANOVA table of linear model fitted to weighted Unifrac distance metric at species level, including analysis of multivariate homogeneity of group dispersions

Variable	Sum Sq	Df	F value	*P*-value	R^2	dispersion *P*-value*
Control *vs.* FAP-NOS	0.04	1	1.32	0.20	0.01	0.85
Age	0.04	1	1.35	0.22	0.01	NA
Sex	0.02	1	0.47	0.85	$4.00*10^{-03}$	0.95
Country	0.04	1	1.22	0.25	0.01	0.80
Mode of delivery	0.01	1	0.45	0.86	$4.00*10^{-03}$	0.80
Main infant diet	0.06	2	0.94	0.48	0.02	0.44
Preterm birth	0.02	1	0.51	0.81	$4.00*10^{-03}$	0.68
Early life traumatic events	0.02	1	0.54	0.79	$4.00*10^{-03}$	0.44
Early life antibiotics treatment	0.03	1	0.89	0.46	0.01	0.85
Probiotics treatment in the last three months	0.02	1	0.72	0.60	0.01	0.80
Lactose specific diet	0.03	2	0.42	0.94	0.01	0.80
PEG	0.04	1	1.18	0.31	0.01	0.88
PPI	0.03	1	1.03	0.37	0.01	0.80
Fecal dry weight	0.04	1	1.17	0.27	0.01	NA
Log_{10} 16S rRNA gene copies per g dry weight	0.05	1	1.55	0.16	0.01	NA
De novo enterotype	0.66	1	21.25	$1.00*10^{-03}$	0.17	0.85
Fecal pH	0.05	1	1.67	0.11	0.01	NA
Residuals	2.34	75	NA	NA	0.61	NA

*FDR-adjusted; Df: degrees of freedom; NA: not applicable; PEG: polyethylene glycol; PPI: proton pump inhibitors; Sum Sq: sum of squares.

Supplementary Table 9. ANOVA table of multiple linear regression model used to explore the *Firmicutes*/*Bacteroidetes* ratio

Variable	Sum Sq	Df	F value	*P*-value	R^2
Control *vs.* FAP-NOS	0.73	1	1.59	0.21	0.01
Age	0.05	1	0.11	0.74	$8.68*10^{-04}$
Sex	0.41	1	0.89	0.35	0.01
Country	0.02	1	0.04	0.85	$2.88*10^{-04}$
Mode of delivery	0.01	1	0.02	0.89	$1.46*10^{-04}$
Main infant diet	0.39	2	0.42	0.66	0.01
Preterm birth	0.39	1	0.86	0.36	0.01
Early life traumatic events	0.08	1	0.18	0.68	$1.43*10^{-03}$
Early life antibiotics treatment	0.00	1	0.00	0.95	$2.65*10^{-05}$
Probiotics treatment in the last three months	0.72	1	1.56	0.22	0.01
Lactose specific diet	0.71	2	0.77	0.47	0.01
PEG	0.14	1	0.30	0.59	$2.42*10^{-03}$
PPI	0.00	1	0.00	0.97	$8.98*10^{-06}$
Fecal dry weight	0.25	1	0.55	0.46	$4.41*10^{-03}$
Log_{10} 16S rRNA gene copies per g dry weight	0.02	1	0.04	0.85	$3.09*10^{-04}$
De novo enterotype	16.08	1	35.09	$8.89*10^{-08}$	0.28
Fecal pH	2.36	1	5.14	0.03	0.04
Residuals	34.36	75	NA	NA	0.61

Df: degrees of freedom; NA: not applicable; PEG: polyethylene glycol; PPI: proton pump inhibitors; Sum Sq: sum of squares.

Supplementary Table 10. ANOVA table of multiple linear regression model used to explore the *Faecalibacterium*/*Bacteroides* ratio

Variable	Sum Sq	Df	F value	*P*-value	R^2
Control *vs.* FAP-NOS	0.25	1	0.46	0.50	$3.56*10^{-03}$
Age	0.23	1	0.42	0.52	$3.23*10^{-03}$
Sex	0.09	1	0.17	0.68	$1.30*10^{-03}$
Country	0.02	1	0.04	0.85	$2.75*10^{-04}$
Mode of delivery	0.06	1	0.10	0.75	$8.07*10^{-04}$
Main infant diet	4.02	2	3.66	0.03	0.06
Preterm birth	1.31	1	2.38	0.13	0.02
Early life traumatic events	0.00	1	0.00	0.97	$1.14*10^{-05}$
Early life antibiotics treatment	0.03	1	0.06	0.82	$4.25*10^{-04}$
Probiotics treatment in the last three months	0.93	1	1.69	0.20	0.01
Lactose specific diet	0.51	2	0.47	0.63	0.01
PEG	0.09	1	0.16	0.69	$1.23*10^{-03}$
PPI	0.73	1	1.32	0.25	0.01
Fecal dry weight	5.32	1	9.69	$2.62*10^{-03}$	0.07
Log_{10} 16S rRNA gene copies per g dry weight	0.15	1	0.27	0.61	$2.07*10^{-03}$
De novo enterotype	16.02	1	29.18	$7.47*10^{-07}$	0.22
Fecal pH	0.28	1	0.52	0.47	0.00
Residuals	41.18	75	NA	NA	0.58

Df: degrees of freedom; NA: not applicable; PEG: polyethylene glycol; PPI: proton pump inhibitors; Sum Sq: sum of squares.
